# A dual role of AcSDKP in chronic kidney disease: Targeting pericyte-driven fibrosis and vascular repair via *VEGF/FGFR1* while eliciting a compensatory *FGFR1* upregulation in apoptotic tubular cells

**DOI:** 10.1371/journal.pone.0355409

**Published:** 2026-09-11

**Authors:** Shiyi Zhu, Lingli Shi, Linlin Huang, Ningxun Cui, Ruyue Chen, Lu Jiang, Annan Zhang, Yunyun Xu, Xiaozhong Li

**Affiliations:** 1 Kidney Pathology and Immunology Laboratory, Pediatric Clinical Research Institute of Soochow University, Pediatric Clinical Medical College of Soochow University, Children’s Hospital of Soochow University, Soochow, China; 2 Pediatric Department, Suzhou Municipal Hospital & Department of Nephrology and Immunology, Children’s Hospital of Soochow University, Soochow, China; 3 Kidney Pathology and Immunology Laboratory, Pediatric Clinical Research Institute of Soochow University, Pediatric Clinical Medical College of Soochow University, Children’s Hospital of Soochow University, Soochow, China; 4 Kidney Pathology and Immunology Laboratory, Pediatric Clinical Research Institute of Soochow University, Pediatric Clinical Medical College of Soochow University, Children’s Hospital of Soochow University, Soochow, China; 5 Department of Nephrology and Immunology, Children’s Hospital of Soochow University, Soochow, China; 6 Department of Nephrology and Immunology, Children’s Hospital of Soochow University, Soochow, China; 7 Electronic Information School of Wuhan University, Wuhan, China; 8 Pediatric Clinical Research Institute of Soochow University, Pediatric Clinical Medical College of Soochow University, Children’s Hospital of Soochow University, Soochow, China; 9 Pediatric Clinical Research Institute of Soochow University, Pediatric Clinical Medical College of Soochow University, Department of Nephrology and Immunology, Children’s Hospital of Soochow University, Soochow, China; Toho University: Toho Daigaku, JAPAN

## Abstract

**Background:**

Renal interstitial fibrosis and peritubular capillary (PTC) rarefaction drive the progression of chronic kidney disease (CKD). While *N-acetyl-seryl-aspartyl-lysyl-proline* (AcSDKP) is an endogenous anti-fibrotic peptide, its specific effects on pericyte-endothelial crosstalk and tubular epithelial survival remain controversial.

**Methods:**

We employed a self-controlled unilateral aristolochic acid (AA)-induced CKD mouse model to evaluate the time-sensitive efficacy of AcSDKP. In vitro, primary murine pericytes, human tubular epithelial cells (HK-2), and endothelial cells were utilized. Functional mechanisms were dissected using VEGF ELISA, RNA-sequencing, and targeted *FGFR1* siRNA knockdown.

**Results:**

In vitro, AcSDKP potently suppressed TGF-β1-induced pericyte-to-myofibroblast transition (PMT) and robustly restored pericyte-derived *VEGF* secretion, thereby rescuing endothelial tubulogenesis. In vivo, early AcSDKP intervention (Week 3) significantly mitigated fibrosis, preserved capillary integrity, and improved systemic renal function (BUN/SCr), whereas late intervention (Week 4) was less effective. Transcriptomics identified the *VEGF/FGFR1* axis as a primary target of AcSDKP. However, in AA-injured HK-2 cells, AcSDKP paradoxically exacerbated apoptosis despite upregulating *FGFR1*. Subsequent siRNA knockdown revealed that silencing *FGFR1* drastically worsened apoptosis, proving that endogenous *FGFR1* upregulation is a compensatory survival response. AcSDKP’s epithelial toxicity is fundamentally *FGFR1*-independent, driven instead by its concurrent inhibition of the Akt survival pathway.

**Conclusion:**

AcSDKP exerts a compartment-specific dual role in CKD. It is a potent vascular protector that halts PMT and promotes *VEGF*-driven endothelial repair, but acts as a pro-apoptotic stressor in severely damaged tubules. Its clinical translation necessitates early-stage intervention and optimized delivery strategies to maximize vascular benefits while mitigating epithelial toxicity.

## 1. Introduction

Chronic kidney disease (CKD) represents a major global public health challenge, frequently progressing to end-stage renal disease [1]. The histological hallmarks of progressive CKD are renal interstitial fibrosis (RIF) and peritubular capillary (PTC) rarefaction [2,3]. Within the renal microenvironment, pericytes, which are perivascular mesenchymal cells responsible for stabilizing endothelial cells, are increasingly recognized as the primary source of scar-forming myofibroblasts [4–6]. Upon renal injury, pericytes detach from the capillary wall and undergo pericyte-to-myofibroblast transition (PMT). This transdifferentiation triggers a “double hit” to the kidneys: it directly drives extracellular matrix deposition while simultaneously destabilizing the endothelium, leading to irreversible capillary dropout and subsequent hypoxia [7,8]. Halting PMT is therefore considered a critical therapeutic target for preserving both the interstitial and vascular compartments.

*N-acetyl-seryl-aspartyl-lysyl-proline* (AcSDKP) is an endogenous, ubiquitously distributed tetrapeptide known for its potent anti-fibrotic and anti-inflammatory properties [9,10]. Previous studies have demonstrated that AcSDKP effectively attenuates renal fibrosis by inhibiting endothelial-to-mesenchymal transition (EndMT) and suppressing macrophage infiltration [6,11]. However, despite its known vascular benefits, it remains largely unexplored whether AcSDKP can directly regulate pericyte behavior by preventing PMT and restoring the critical pericyte-endothelial crosstalk required for angiogenesis.

Furthermore, the mechanisms underlying the effects of AcSDKP in different renal compartments, particularly in the tubular epithelium under toxic stress, are highly complex. Fibroblast growth factor receptor 1 (*FGFR1*) plays a vital role in cellular survival and tissue repair. [12,13] While *FGFR1* activation is generally considered protective against tubular injury [14,15], its precise mechanistic role in aristolochic acid (AA)-induced tubular apoptosis remains ambiguous. The potential for AcSDKP to modulate this specific survival pathway necessitates rigorous functional dissection.

Therefore, to address these critical gaps, the present study aims to: (1) determine whether AcSDKP mitigates RIF by inhibiting PMT and restoring vascular repair via the *VEGF/FGFR1* paracrine axis; (2) evaluate the time-sensitive therapeutic window of AcSDKP using a novel, self-controlled in vivo AA-induced CKD mouse model; and (3) functionally dissect the controversial role of endogenous *FGFR1* in tubular epithelial apoptosis using targeted gene knockdown, thereby clarifying the dual nature of AcSDKP in the progression of CKD.

## 2. Materials and methods

### 2.1. Animal models and experimental design

All surgery was performed under 1% sodium pentobarbital anesthesia (40 mg/kg, intraperitoneally). For sacrifice, mice were anesthetized with sodium pentobarbital to induce a deep unconscious state, followed by blood collection via cardiac puncture. Death was subsequently confirmed by cervical dislocation. All efforts were made to minimize suffering.

Male C57BL/6 mice (8 weeks old, 20–25 g) were purchased from the Animal Experiment Center of Soochow University. To comprehensively evaluate both the natural progression of fibrosis and the therapeutic efficacy of AcSDKP, two distinct sets of in vivo experiments were designed. In both models, disease was induced via a single intraperitoneal injection of aristolochic acid I (AAI, 5 mg/kg; A5512, Sigma-Aldrich, St. Louis, MO, USA).

A. Time-Course Progression Model of AAN: To establish the baseline progression of AAI-induced renal fibrosis, a cohort of mice was monitored following the single AAI injection. Body weights were recorded weekly. Subgroups of mice were subsequently sacrificed at Weeks 1, 2, 3, and 4 post-injection. Kidney tissues were harvested and subjected to Hematoxylin and Eosin (H&E) and Masson’s trichrome staining, as well as immunohistochemical staining for α-SMA and PDGFRβ, to systematically evaluate the dynamic degree of interstitial fibrosis over time.
B. Self-Controlled Unilateral Intervention Model: Based on the progression timeline, we sought to determine the time-sensitive therapeutic window of AcSDKP. To eliminate inter-individual genetic and baseline variability, a novel self-controlled unilateral model was established using a separate cohort of AAI-injected mice:
  a) Pre-treatment Control (Baseline): At Week 3 (early stage) or Week 4 (late stage) post-AAI injection, mice were anesthetized, and a left nephrectomy was surgically performed. The harvested left kidney served as the exact “Pre-treatment” fibrotic baseline for that specific animal.
  b) Treatment Phase: Following a 24-hour post-surgical recovery period, mice were administered either AcSDKP (1 mg/kg/day; HY-P0266, MedChemExpress, USA) or an equal volume of saline via daily intraperitoneal injection for 4 consecutive weeks.
  c) Post-treatment Endpoint and Blood Collection: At the conclusion of the 4-week treatment period (i.e., Week 7 for the early-intervention group and Week 8 for the late-intervention group), the remaining right kidney was harvested as the “Post-treatment” sample to evaluate fibrotic reversal.

To dynamically monitor systemic Blood Urea Nitrogen (BUN) and serum creatinine (SCr) levels, blood samples were collected at two specific time points using ethically approved methods. At the time of the initial left nephrectomy (Pre-treatment baseline), survival blood collection was performed via the tail vein under anesthesia. At the final sacrifice (Post-treatment endpoint), terminal blood collection was performed via retro-orbital bleeding under deep sodium pentobarbital anesthesia prior to cervical dislocation. Serum was subsequently separated by centrifugation (3000 rpm, 15 min) for biochemical analysis.

### 2.2. Cell culture and treatments

Primary Pericytes: Mouse renal pericytes were isolated using immunomagnetic beads and cultured in Pericyte Medium (PriMed-iCell-015, Icell Bioscience Inc., Shanghai, China).

HK-2 Cells: Human proximal tubular epithelial cells were obtained from ATCC (PCS-400–010) and cultured in DMEM/F12 medium (11320033, Gibco, USA) supplemented with 10% fetal bovine serum (FBS, 10100, Australia, Gibco).

bEnd.3 Cells: Mouse brain microvascular endothelial cells were obtained from Procell (CP-M108, Procell, China) and cultured in DMEM. Cells were maintained at 37°C in a humidified 5% CO2 atmosphere.

For in vitro injury models, pericytes were stimulated with TGF-β1 10 ng/mL (HY-P70648, MedChemExpress, USA) and HK-2 cells were treated with Aristolochic Acid 10 μg/mL (AA, A5512, Sigma-Aldrich, USA). AcSDKP (100 nM, HY-P0266, MedChemExpress, USA) was added 120 min prior to or concurrently with stimulation as indicated.

### 2.3. Cell apoptosis

HK-2 cells were seeded in 6-well plates (2 × 105 cells/well) and synchronized in FBS-free DMEM/F12 for 12 h before treatment. Cells were divided into 5 groups (n = 3 each group).

Group A (Control): FBS-free DMEM/F12 for 24 h.

Group B (AA): 10 μg/mL aristolochic acid (AA, A5512, Sigma-Aldrich, USA) in FBS-free medium for 24h.

Group C (AA + AcSDKP): 10⁴ nM AcSDKP pretreatment for 2h, followed by 10 μg/mL AA for 24h.

Group D (AA + AcSDKP+PD173074): 100 nM PD173074 (*FGFR1* inhibitor, S1264, Selleck, USA) + 10⁴ nM AcSDKP pretreatment for 2h, then 10 μg/mL AA for 24 h.

Group E (AA + AcSDKP+FGF2): 100 ng/mL FGF2 (*FGFR1* ligand, HY-P7004, MedChemExpress) + 10⁴ nM AcSDKP pretreatment for 2h, then 10 μg/mL AA for 24 h.

### 2.4. Cell apoptosis (Flow Cytometry)

Cell apoptosis detection was performed in accordance with the instructions of the PE Annexin V Apoptosis Detection Kit. Briefly, target cells were first collected and resuspended in pre-chilled PBS for washing 2 times. Subsequently, cells were resuspended in 1 × Binding Buffer to a final concentration of 1 × 10⁶ cells/mL. Next, 100 μL of the cell suspension (containing 1 × 105 cells) was transferred to a flow cytometry tube, followed by the addition of 5 μL PE Annexin V and 5 μL 7-AAD. The cells were gently mixed and incubated at 25°C in the dark for 15 minutes. Finally, 400 μL of 1 × Binding Buffer was added to each tube, and flow cytometric analysis was completed within 1 hour. Cells were categorized into two subsets: early apoptosis (PE Annexin V-positive, 7-AAD-negative) and late apoptosis (PE Annexin V-positive, 7-AAD-positive).

### 2.5. Small interfering RNA (siRNA) transfection

To verify the role of *FGFR1*, HK-2 cells were transfected with *FGFR1*-specific siRNA or negative control (NC) siRNA (HY-RS04911, MedChemExpress, NJ, USA). Transfection was performed using siRNA Transfection Reagent (HY-K2017, MedChemExpress, NJ, USA) according to the manufacturer’s instructions. Knockdown efficiency was verified by RT-qPCR and Western blot 24–48 h post-transfection.

### 2.6. ELISA for VEGF secretion

To evaluate paracrine signaling, VEGF levels in pericyte culture supernatants were quantified using a Mouse VEGF ELISA Kit (Yamay, Cat# R014768, China). Supernatants were collected from Control, TGF-β1, and TGF-β1 + AcSDKP groups. The assay was performed following the manufacturer’s protocol. Optical density (OD) was measured at 450 nm using a microplate reader (Varioskan LUX, Thermo Scientific™, USA).

### 2.7. Western blot analysis

Kidney tissues or cells were lysed in RIPA buffer (89900, Thermo Scientific™, USA) containing protease and phosphatase inhibitors (78440, Thermo Scientific™, USA). Protein concentration was determined using a BCA kit (ab102536, Abcam, USA). Equal amounts of protein were separated by SDS-PAGE (T10420LGe, ACE Biotechnology, China) and transferred onto PVDF membranes (88518, Thermo Scientific™, USA). Membranes were blocked and incubated overnight at 4°C with the following primary antibodies:

Anti-Caspase-3/p17/p19 (1:10000, Cat# 82202–1-RR, Proteintech, China) – detects both pro- and cleaved-caspase 3

Anti-Bax (1:2000, Cat# HY-P80028, MedChemExpress, NJ, USA)

Anti-Bcl-2 (1:2000, Cat# bs-0230R, Proteintech, China)

Anti-Phospho-Akt (1:1000, Cat# 9271T, CST, USA)

Anti-Akt (1:1000, Cat# EPR16798, Abcam, USA)

Anti-FGFR1 (1:1000, Cat# bs-0230R, Bioss Antibodies, USA)

Anti-GAPDH (1:50000, Cat# 60004–1-Ig, Proteintech, China)

Anti-PDGFRβ (1:1000, Cat# 3169T, CST, USA)

Anti-α-SMA (1 μg/mL, Cat# AB7817, Abcam, USA)

Anti-CD31 (1:1000, AB222783, Abcam, USA)

Secondary antibodies (HRP-conjugated Goat Anti-Rabbit/Mouse IgG, Ab6721, Ab6789, Abcam, USA) were applied for 1 h at room temperature. Bands were visualized using an ECL detection system (34580, Thermo Scientific™, USA) and quantified using ImageJ software.

### 2.8. Quantitative real-time PCR (RT-qPCR)

Total RNA was extracted using Trizol reagent (15596026CN, Invitrogen™, USA). cDNA was synthesized using a Reverse Transcription Kit (RR037A, Takara, Japan). qPCR was performed using SYBR Green Master Mix (KCQS02, Sigma-Aldrich, USA) on a Real-Time PCR System (LightCycler 480 Instrument II®). Primer sequences are listed in Table 1. Relative gene expression was calculated using the 2^-ΔΔCt method.

**Table 1:** Primer sequences of RT-qPCR.

Species	Name	Forward primer (5’ → 3’)	Reverse primer (5’ → 3’)
Human	*FGFR1*	GACTCCGGCCTCTATGCTTG	TATGGAGCTACGGGCATACG
Human	*GAPDH*	AATGGGCAGCCGTTAGGAAA	GCCCAATACGACCAAATCAGAG
Mouse	*Gapdh*	GAGCCTCCTCCAATTCAACCC	TTGTCTACGGGACGAGGAAAC
Mouse	*Il-6*	TAGTCCTTCCTACCCCAATTTCC	TTGGTCCTTAGCCACTCCTTC
Mouse	*Tgf-β1*	CTCCCGTGGCTTCTAGTGC	GCCTTAGTTTGGACAGGATCTG
Mouse	*Pdgfrβ*	TTCCAGGAGTGATACCAGCTT	AGGGGGCGTGATGACTAGG
Mouse	*Desmin*	GTGGATGCAGCCACTCTAGC	TTAGCCGCGATGGTCTCATAC
Mouse	*α-sma*	TGGCACCACTCTTTCTATAACG	GGTCATTTTCTCCCGGTTGG
Mouse	*Vimentin*	CGGCTGCGAGAGAAATTGC	CCACTTTCCGTTCAAGGTCAAG
Mouse	*Hif-1α*	ACCTTCATCGGAAACTCCAAAG	ACTGTTAGGCTCAGGTGAACT
Mouse	*Vegfr*	GCACATAGAGAGAATGAGCTTCC	CTCCGCTCTGAACAAGGCT

### 2.9. Histology and immunofluorescence

Kidney tissues were fixed in 4% paraformaldehyde and embedded in paraffin. Sections (4 μm) were stained with Masson’s trichrome to assess fibrosis. For immunofluorescence, sections were incubated with primary antibodies against CD31(1/150, AB222783, Abcam, USA), PDGFRβ (1/100, 3169T, CST, USA), or α-SMA (1/100, AB7817, Abcam, USA), followed by fluorophore-conjugated secondary antibodies. Nuclei were stained with DAPI. Images were captured using a fluorescence microscope (Olympus BX53, Japan).

### 2.10. Bioinformatics and RNA-sequencing

Total RNA was extracted from kidney tissues (n = 3 per group). RNA integrity was assessed using the Agilent 2100 Bioanalyzer. Library preparation and sequencing were performed on the Illumina NovaSeq 6000 platform.

### 2.11. Analysis

Differentially Expressed Genes (DEGs) were identified using DESeq2 with a cutoff of |log2FoldChange| > 1 and P-value < 0.05. KEGG pathway enrichment and Gene Set Enrichment Analysis (GSEA) were performed to identify significantly altered signaling pathways.

### 2.12. Statistical analysis

Data are presented as mean ± standard deviation (SD) from at least three independent experiments. Normality of the data distribution was assessed using the Shapiro-Wilk test. For comparisons between two independent groups, an unpaired two-tailed Student’s t-test was performed. For comparisons among three or more groups, a One-way Analysis of Variance (ANOVA) followed by Tukey’s post hoc test was employed. In cases where the data did not pass the normality test, the non-parametric Mann-Whitney U test (for two groups) or Kruskal-Wallis test followed by Dunn’s multiple comparisons test (for multiple groups) was utilized. All statistical analyses and graph generation were performed using GraphPad Prism 9.0 software (GraphPad Software, San Diego, CA, USA). A *P*-value < 0.05 was considered statistically significant.

## 3. Results

### 3.1. AcSDKP Attenuates the differentiation of pericytes into myofibroblasts

**Fig 1:**
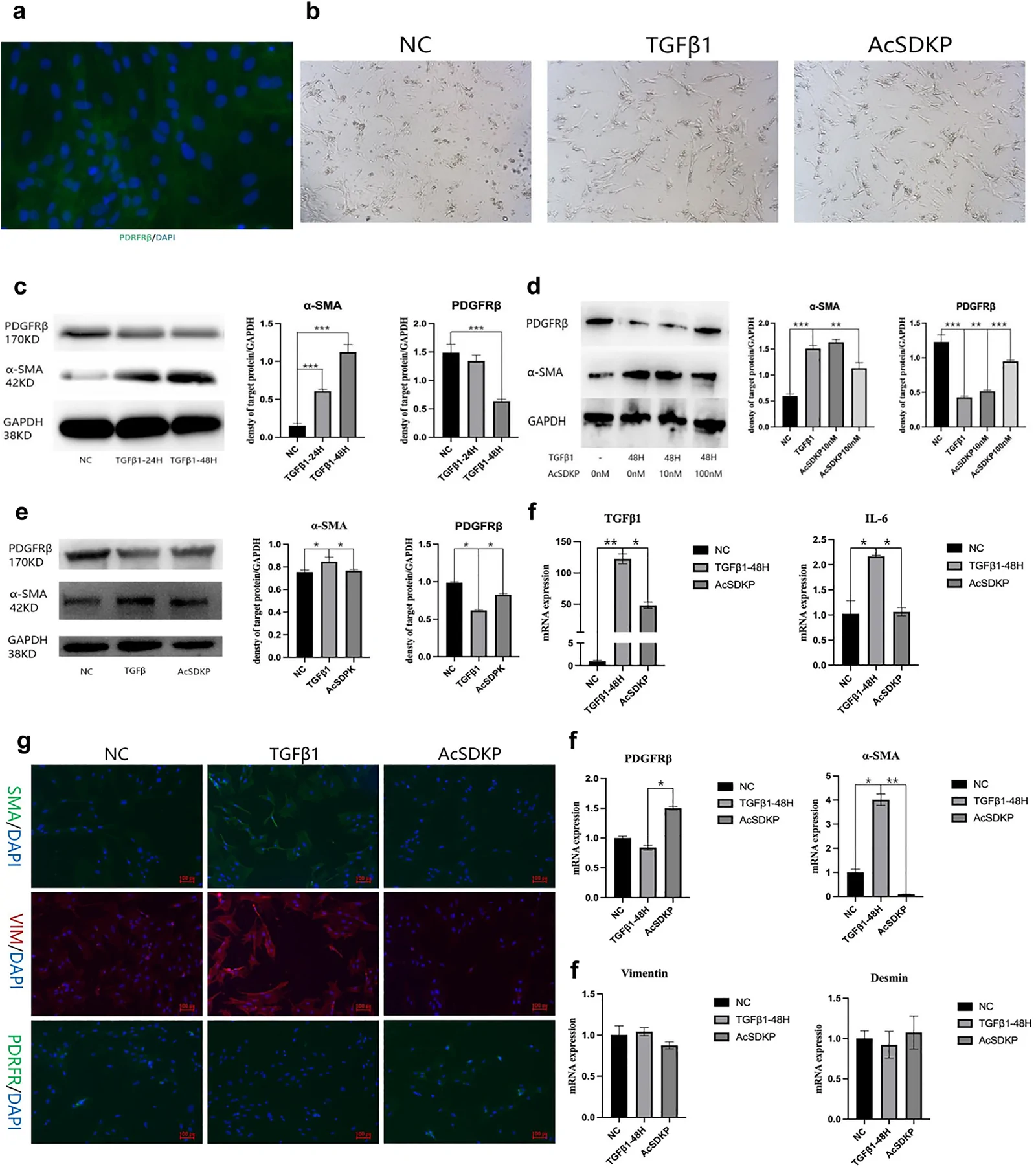
(a) Primary pericytes were characterized by dual-channel fluorescent staining, with the representative microscopic field captured under 100× optical magnification. (b) Altered primary pericytes morphology under white light, 100× magnification. (c)–(e) Protein expression levels of *α-SMA* and *PDGFRβ* detected by Western blot and gray value analyses (n=3). The results are expressed as mean±standard deviation, *P < 0.05; **P < 0.01; ***P < 0.001. (f) Pericyte–myofibroblast transdifferentiation and changes in the expression of relevant primers after AcSDKP intervention treatment. n = 3. Results are expressed as mean±standard deviation. *P < 0.05; **P < 0.01. (g) Immunofluorescence triple-labeling demonstrates *α-SMA, Vimentin,* and *PDGFRβ* co-expression within pericyte populations captured at 100× optical magnification.

*PDGFRβ*, a pericyte surface marker, was identified based on microscopic cell morphology and cellular immunofluorescence (Fig 1a). White light microscopy revealed that TGFβ1-stimulated pericytes underwent a significant morphological shift, differentiating into spindle-like myofibroblasts. AcSDKP effectively prevented this transformation, restoring the myofibroblasts back to a pericyte morphology (Fig 1b).

Following stimulation with 10 ng/mL TGFβ1 for 24 and 48 hours, cell proteins were collected and α-SMA and PDGFRβ levels measured in each group. TGFβ1 (10 ng/mL) significantly altered expression levels after 48 hours compared to the 24-hour group (Fig 1c). After 48 hours of TGFβ1 (10 ng/mL) stimulation, both groups received different concentrations of AcSDKP (10 or 100 nM) for 24 hours. The results demonstrated that 100 nM AcSDKP exerted a significantly stronger intervention effect than 10 nM AcSDKP. Consequently, the subsequent in vitro cellular experiments employed TGFβ1 (10 ng/mL) for 48 hours followed by AcSDKP (100 nM) for 24 hours (Fig 1d). Based on these preliminary conditions, cells were divided into three groups: control, TGFβ1, and TGFβ1 + AcSDKP. Proteins extracted from all three groups underwent Western blot analysis to detect α-SMA and PDGFRβ expression. α-SMA levels in the TGFβ1 group exceeded those in the control group. AcSDKP intervention reduced α-SMA levels in the TGFβ1 + AcSDKP group compared to the TGFβ1 group. PDGFRβ exhibited the opposite trend, being lower in the TGFβ1 group and higher following AcSDKP treatment (Fig 1e).

The RT-PCR results revealed that *α-SMA, IL-6*, and *TGFβ1* expression in the TGFβ1 group significantly exceeded that in the control group (*P* < 0.05). *Vimentin* levels in the TGFβ1 group were elevated compared to the control group, whereas *Desmin* expression trended lower. However, these latter differences lacked statistical significance. Notably, AcSDKP significantly suppressed the expressions of *α-SMA, IL-6, TGFβ1*, and *Vimentin* (Fig 1f).

Myofibroblast levels were evaluated based on *α-SMA* and *Vimentin* level, while pericyte activity was assessed via *PDGFRβ* level. The results demonstrated that TGFβ1 stimulation of pericytes triggered a marked increase in *α-SMA* and *Vimentin* expression coupled with a decrease in *PDGFRβ* levels. Following AcSDKP intervention, however, both *α-SMA* and *Vimentin* levels substantially declined, while *PDGFRβ* expression rebounded, highlighting the therapeutic potential of AcSDKP in modulating these cellular responses (Fig 1g). Hence, we propose that AcSDKP effectively reduces pericyte-to-fibroblast transformation in vitro. Furthermore, AcSDKP demonstrably induces neovascularization, both in vitro and in vivo [16].

### 3.2. AcSDKP ameliorates vascular dysfunction caused by pericyte-to-myofibroblast transition

**Fig 2:**
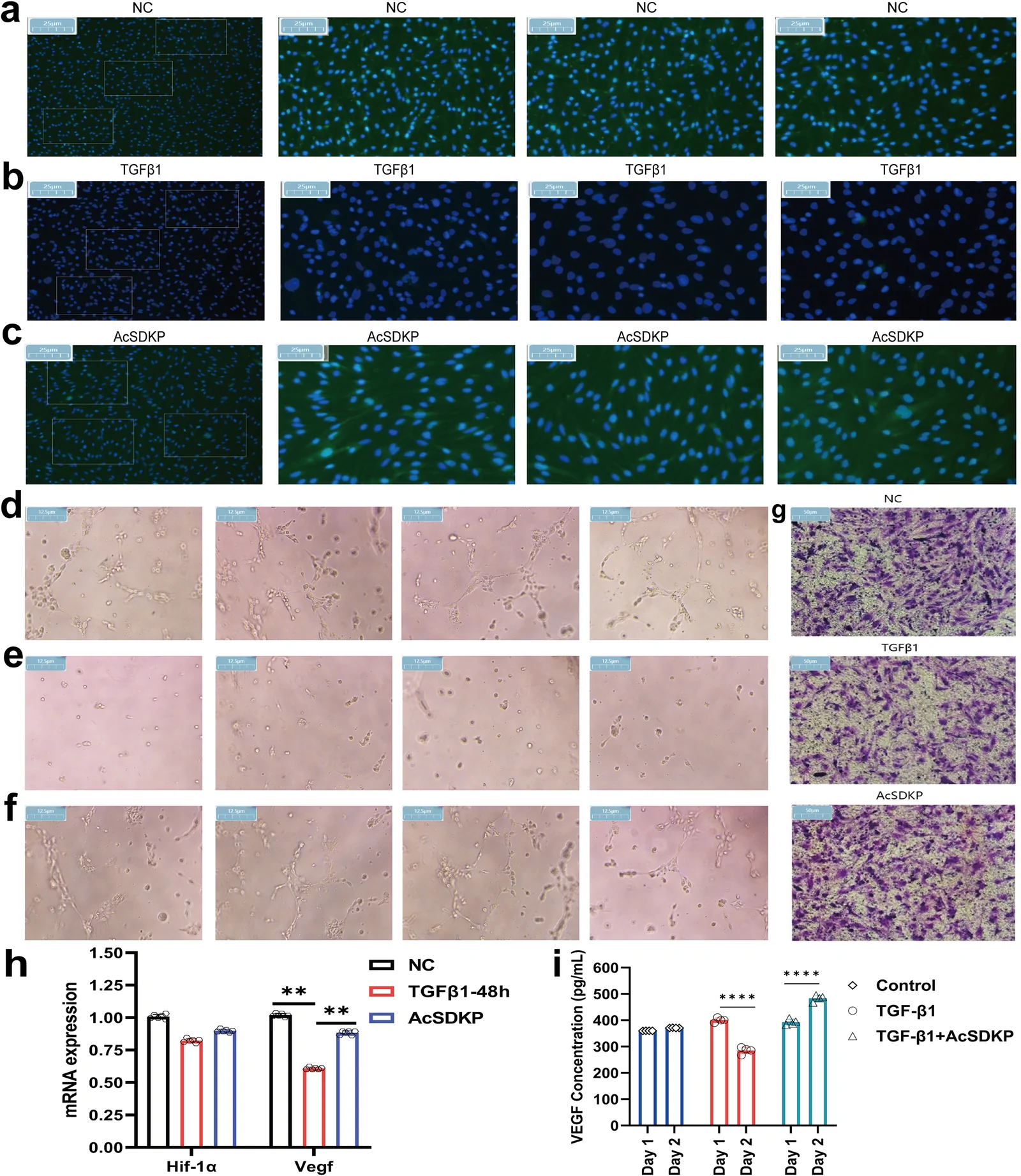
AcSDKP ameliorates vascular dysfunction caused by pericyte–myofibroblast transition. (a)–(c) Immunofluorescence expression of endothelial cells co-cultured with pericytes under microscopy (40 × magnification), with three randomly selected fields of view. Compared with the control group, the *CD31* level was significantly decreased after the co-culture of endothelial cells with TGFβ1-stimulated pericyte supernatant, while the *CD31* level in the AcSDKP intervention group showed partial recovery. (d)–(g) The endothelial cell tube formation assay showed that the TGFβ1 group cells failed to form tubes in contrast to that in the control, while AcSDKP restored this ability. Migration assays revealed weakened endothelial cell migration in the TGFβ1 group compared to that in the control, with AcSDKP restoring the mobility. (h) Changes in the expression of endothelial cell-associated primers in each group after pericyte–endothelial cell co-culture. n = 6. The results are expressed as mean±standard deviation. **P < 0.01. (i) TGFβ1 stimulation significantly impaired the secretory function of pericytes, reducing *VEGF* levels to 282.05 ± 12.7 pg/mL compared to 370.54 ± 4.5 pg/mL in the control group (*P <* 0.0001). AcSDKP treatment robustly restored *VEGF* secretion to 485.81 ± 8.1 pg/mL (*P* < 0.0001 vs. TGFβ1 group).

Detection of CD31 serves as an indicator of endothelial cell neovascularization levels. In contrast to the control group (Fig 2a), CD31 expression dramatically decreased within endothelial cells co-cultured with TGFβ1-stimulated pericyte supernatant (Fig 2b). However, CD31 levels were markedly restored in the AcSDKP intervention group (Fig 2c).

After plating cells on the matrix gel for 4 hours and examining them under the microscope, we observed a stark contrast to the control group (Fig 2d): endothelial cells in the TGFβ1 group failed to form any tubes (Fig 2e), while those treated with AcSDKP regained their tube-forming capability (Fig 2f). Furthermore, endothelial cell migration was markedly impaired in the TGFβ1 group compared to the control, yet AcSDKP treatment fully restored this migratory capacity (Fig 2g).

Real-time PCR analysis demonstrated that *VEGF* expression plunged significantly in the TGFβ1 group relative to controls but surged significantly in the AcSDKP group. Conversely, *Hif-1α* expression displayed only a modest reduction in the TGFβ1 group and a slight elevation in the AcSDKP group, though not statistically significant. While observable trends existed for *Hif-1α* in both groups, the differences lacked statistical significance (Fig 2h). Furthermore, to validate the molecular mechanism of this crosstalk, we quantified VEGF secretion in pericyte culture supernatants using ELISA. As shown in Fig 2i, TGFβ1 stimulation significantly impaired the secretory function of pericytes, reducing VEGF levels to 282.05 ± 12.7 pg/mL compared to 370.54 ± 4.5 pg/mL in the control group (*P* < 0.0001). Notably, AcSDKP treatment robustly restored VEGF secretion to 485.81 ± 8.1 pg/mL (*P* < 0.0001 vs. TGFβ1 group). These data provide evidence that AcSDKP exerts its pro-angiogenic effects by reprogramming pericytes to secrete VEGF.

### 3.3. AcSDKP mitigates renal interstitial fibrosis in AA-induced CKD self-controlled mouse model

**Fig 3:**
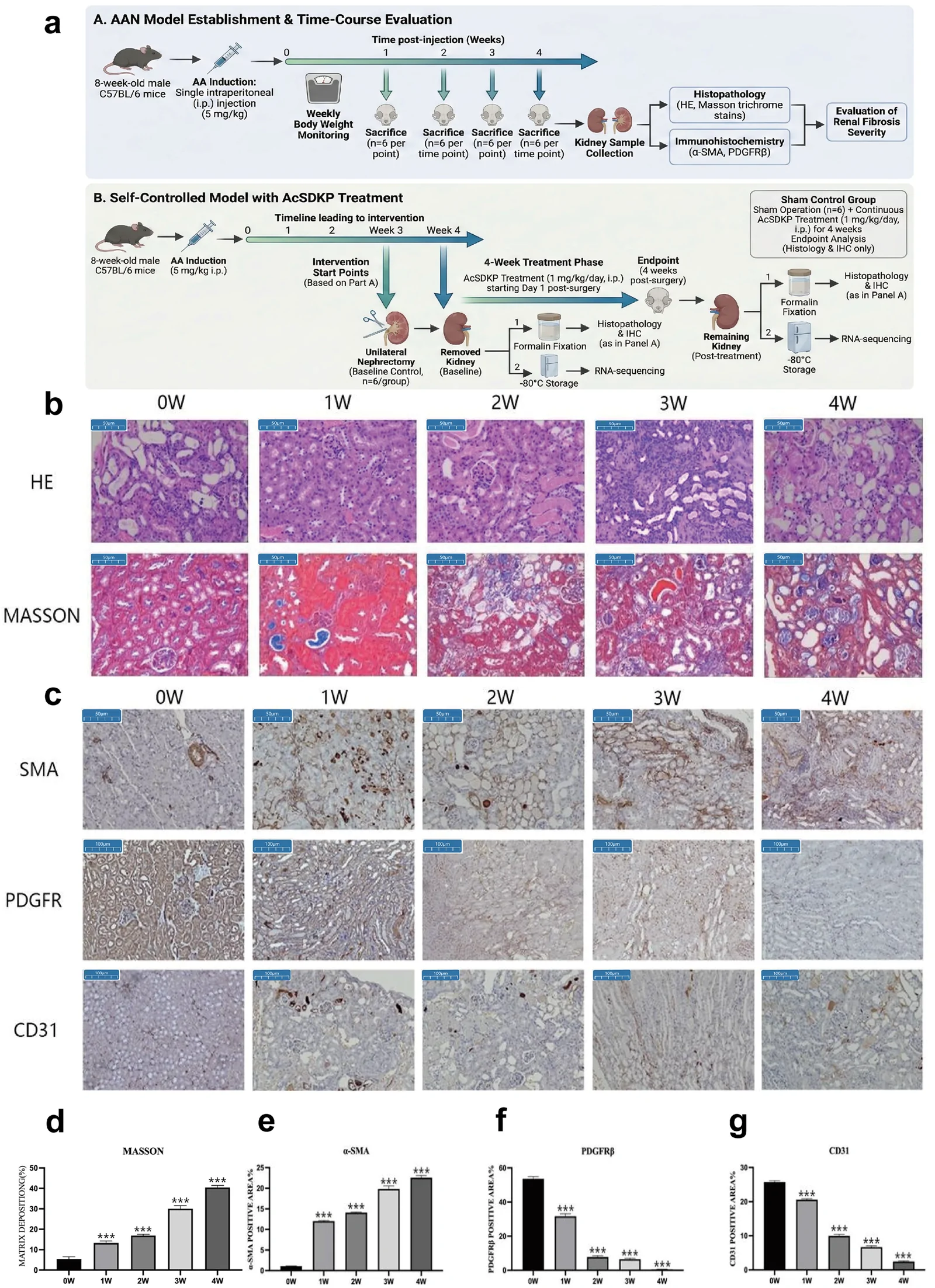
Mouse aristolochic acid nephropathy model construction experiment results: (a)Flowchart of the experimental procedure for constructing a mouse aristolochic acid nephropathy model. (b)Results of H&E and MASSON staining. Four images were randomly selected from different areas of the paraffin sections. The results are expressed as mean ± standard deviation; ***P < 0.001, with 0 w. (c) Immunohistochemical staining results of weekly renal pathological changes in 8-week-old male C57BL/6 mice after modeling with intraperitoneal injection of AA. (d)–(f) Randomly selected four images from different areas of the paraffin sections. The expression of *α-SMA, PDGFRβ*, and *CD31* were analyzed and statistically evaluated using ImageJ. The results were presented as mean ± standard deviation; ***P < 0.001, with 0 w.

In contrast to previous research, this study employed distinct nodal approaches for post-modeling intervention. (Fig 3a) Following successful modeling via intraperitoneal injection of aristolochic acid, the unilateral kidney served as the disease group and was subsequently administered AcSDKP for the other kidney of the same single mouse. Pathological alterations observed in the kidneys before and after the self-control experimental model effectively illustrated the amelioration of fibrosis.

H&E staining results revealed proximal tubular epithelial cell swelling, partial necrosis, and casts observable during the 1st week of aristolochic acid nephropathy modeling in mice. Each subsequent week witnessed progressively worsening tubular necrosis and glomerular shrinkage accompanied by hardening, culminating in the 4th week with distal tubular necrosis and lumen expansion (Fig 3b).

As the disease advances and renal tubular necrosis intensifies, the expanse of blue collagen fibers revealed by MASSON staining steadily expands. This progression demonstrates a statistically notable increase each week compared to baseline measurements at week 0. By the 3rd week, collagen fibers account for approximately 30% of the total area, swelling to roughly 40% by the 4th week (Fig 3a, 3b).

Immunohistochemical results revealed that α-SMA expression steadily rises with disease progression. During the 1st week, α-SMA surged significantly compared to the normal group. Expression plateaued in the 2nd week, remaining comparable to week 1. By the 3rd week, fibrosis intensified markedly, followed by a slight increase in the 4th week relative to week 3 (Fig 3c, 3d). As renal fibrosis worsened, PDGFRβ expression progressively diminished, nearing zero by the 4th week (Fig 3c, 3e). CD31 mirrored PDGFRβ’s proportional decline, reaching its lowest point in the 4th week (Fig 3c, 3f).

**Fig 4:**
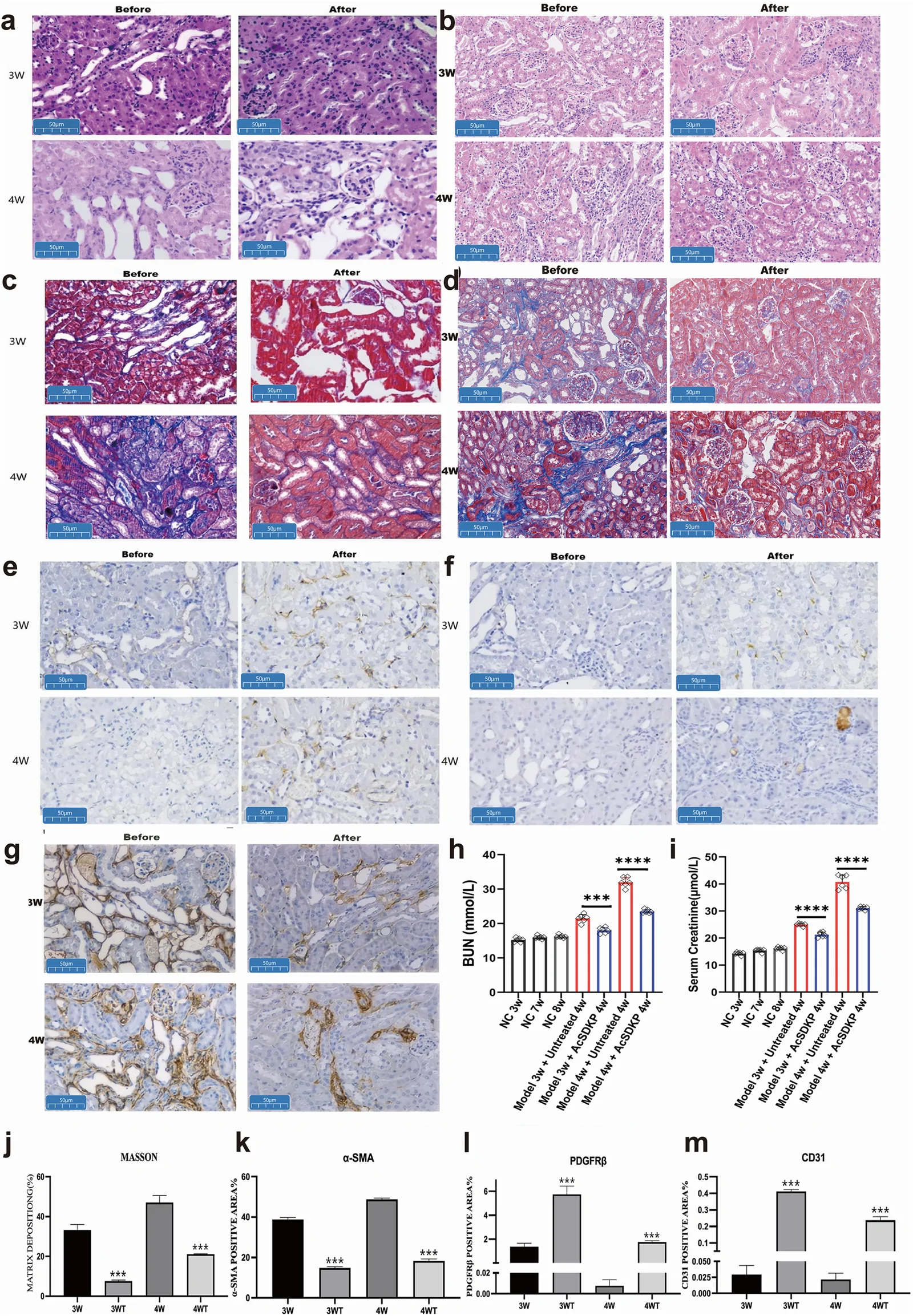
H&E staining, MASSON staining, and immunohistochemistry results obtained after treatment with AcSDKP. (a), (b) H&E staining of mouse aristolochic acid self-control model. (c)–(d), (j) MASSON staining of mouse AA self-control model. Four images were randomly selected from different areas. The MASSON staining results are presented as mean ± standard deviation; ***The 3WT (post-treatment) group compared to the 3W (pre-treatment) group, and the 4WT (post-treatment) group compared to the 4W (pre-treatment) group: *P* < 0.001. (g), (k) *α-SMA* staining of mouse AA self-control model. Four images were randomly selected from different areas, with results expressed as mean ± standard deviation; ***The 3WT group compared with the 3W group, and the 4WT group compared with the 4W group: *P* < 0.001. (e), (l) *PDGFRβ* staining of mouse AA self-control model. Four images were randomly selected from different areas, with the results expressed as mean ± standard deviation; ***The 3WT group compared with the 3W group, and the 4WT group compared with the 4W group: *P* < 0.001. (f), (m) *CD31* staining of mouse AA self-control model. Four images were randomly selected from different areas. The statistics showed the results are expressed as mean ± standard deviation; ***The 3WT group compared with the 3W group, and the 4WT group compared with the 4W group: *P* < 0.001. (h), (i)BUN and SCr levels were measured to evaluate systemic renal function across different experimental stages. The normal control (NC) groups at 3, 7, and 8 weeks demonstrate baseline stability. The untreated AA model groups exhibit an elevation in both BUN and SCr levels over time. Early intervention with AcSDKP (treatment initiated at Week 3) attenuated the AA-induced elevation of BUN and SCr. Late intervention (treatment initiated at Week 4) showed a partial reduction but was less effective than early treatment. Data are presented as mean ± SD (n = 6 per group). Statistical significance was determined by One-way ANOVA followed by Tukey’s post hoc test. **** *P* < 0.0001 versus the corresponding untreated AA model group.

For the AcSDKP treatment group, H&E staining indicated no significant improvement in tubular necrosis before versus after treatment, whether initiated in week 3 or week 4 (Fig 4a, 4b). MASSON results demonstrated a significant reduction in collagen fiber area for both treatment groups post-intervention. Initiating treatment in the 3rd week yielded a markedly more pronounced effect, with a greater decrease in amplitude (Fig 4c, 4d, 4j). Following AcSDKP treatment in weeks 3 and 4, α-SMA expression decreased to varying degrees in both groups (Fig 4g,4k). Treatment prompted a slight elevation in PDGFRβ during weeks 3 and 4 compared to pre-treatment levels in both cohorts. Nevertheless, PDGFRβ failed to restore pre-injury levels; notably, the recovery effect was substantially stronger after week 3 treatment (Fig 4e, 4l). CD31 levels rose in both groups post-treatment, with a more pronounced increase observed following week 3 intervention. However, CD31 levels also remained below pre-injury baselines(Fig 4f, 4m). Consistent with the histological improvements, functional analysis showed that early AcSDKP intervention significantly attenuated the elevation of BUN and serum creatinine levels compared to the untreated AA group(*P < 0.0001*), indicating a recovery of systemic renal filtration function. (Fig 4h,4i)

**Fig 5:**
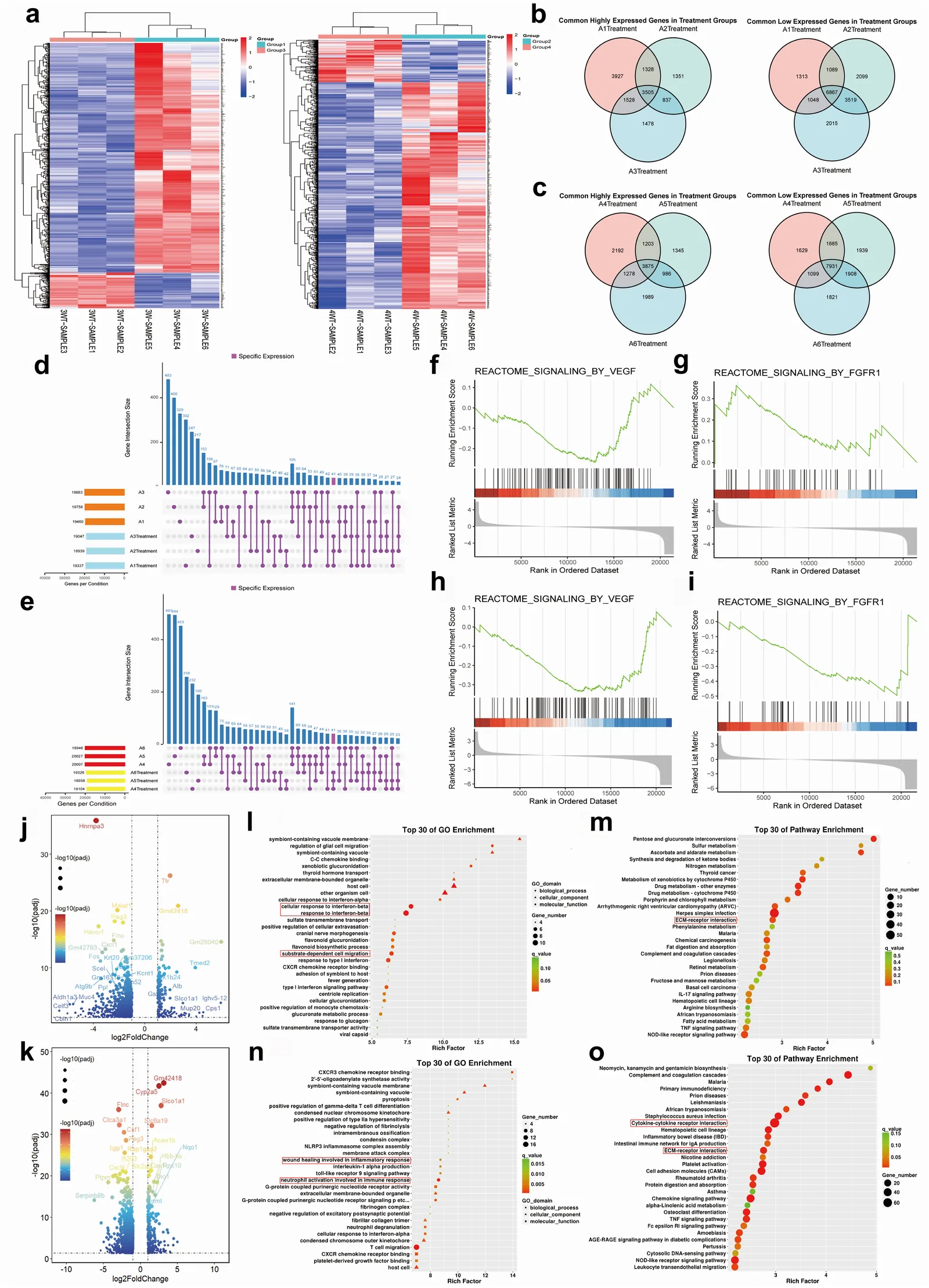
The gene sequencing results of the AcSDKP treatment group 3 and 4 weeks after modeling. (a) Heatmap of RNA sequencing data in the aristolochic acid model at 3 weeks, 4 weeks, and after 4 weeks of treatment with AcSDKP. (b) Through RNA sequencing analysis, the Venn diagram of genes with upregulated or downregulated expression in the aristolochic acid 3-week model group after AcSDKP treatment (n = 3). (c) Venn diagram of genes with upregulated or downregulated expression after 4-week treatment with AcSDKP in the aristolochic acid model group as determined by RNA sequencing (n = 3). (d) The upset plot comparing the AcSDKP treatment group with the aristolochic acid 3-week model group, showing 41 shared treatment group gene expressions (n = 3). (e) The upset plot comparing the AcSDKP treatment group with the aristolochic acid 4-week model group, showing 41 shared treatment group gene expressions (n = 3). (f), (g) GSEA plots of *VEGF* and *FGFR1* in the 3-week model group versus treatment group. (h), (i) GSEA plots of *VEGF* and *FGFR1* in the 4-week model group versus treatment group. (j), (k) Volcano plots of gene expression differences after treatment in the 3-week and 4-week model groups. (l), (m) Three-week model and post-treatment GO and pathway enrichment plots. (n), (o) Four-week model and post-treatment GO and pathway enrichment plots.

To investigate AcSDKP’s role in modulating pericyte-endothelial cell interactions to mitigate renal fibrosis, RNA sequencing was performed on untreated 3-week aristolochic acid model groups (A1, A2, A3), corresponding treated groups (A1T, A2T, A3T), untreated 4-week model groups (A4, A5, A6), and their treated counterparts (A4T, A5T, A6T) (Fig 5a).

Preliminary analysis employing Venn diagrams highlighted commonly upregulated and downregulated gene expression differences across the three samples within each group (Fig 5b, 5c). Compared to pre-treatment levels, the 3-week treatment group exhibited 1040 downregulated genes (including *Hnrnpa3*) and 186 upregulated genes (such as *Ttr* and *Gm42418*) post-treatment (*P* < 0.05, fold change >2.0) (Fig 5j). Similarly, the 4-week treatment group revealed 1480 downregulated genes (e.g., *Flnc*) and 256 upregulated genes (e.g., *Gm42418*, *Cyp2a5*, *Slco1a1*, and *Slc6a19*) after intervention (*P* < 0.05, fold change >2.0) (Fig 5k). Upset analysis uniquely identified 41 genes co-expressed exclusively in the treatment groups at both post-treatment time points (Fig 5d, 5e). GSEA enrichment analysis of these differentially expressed genes demonstrated their primary enrichment within the *VEGF* and *FGFR1* pathways. Post-treatment levels in these signaling pathways showed significant upregulation compared to pre-treatment states (Fig 5f, 5g, 5h, 5i). GO pathway enrichment analysis indicated that type I interferons (like IFN-α/β) exert anti-fibrotic effects by suppressing the *TGFβ/Smad* pathway, thereby reducing ECM production. AcSDKP appears to enhance interferon sensitivity, promoting anti-inflammatory and endothelial protective outcomes (Fig 5l, 5m). ECM–receptor interactions critically regulate cell adhesion, migration, and signaling; their dysregulation drives excessive ECM deposition (including collagen and fibronectin), a core mechanism underlying renal fibrosis. AcSDKP potentially balances inflammation and repair processes, preventing excessive fibrotic scar formation (Fig 5n, 5o).

#### 3.3.1. Validation of the *VEGF/FGFR1* axis and functional dissection of *FGFR1.*

**Fig 6:**
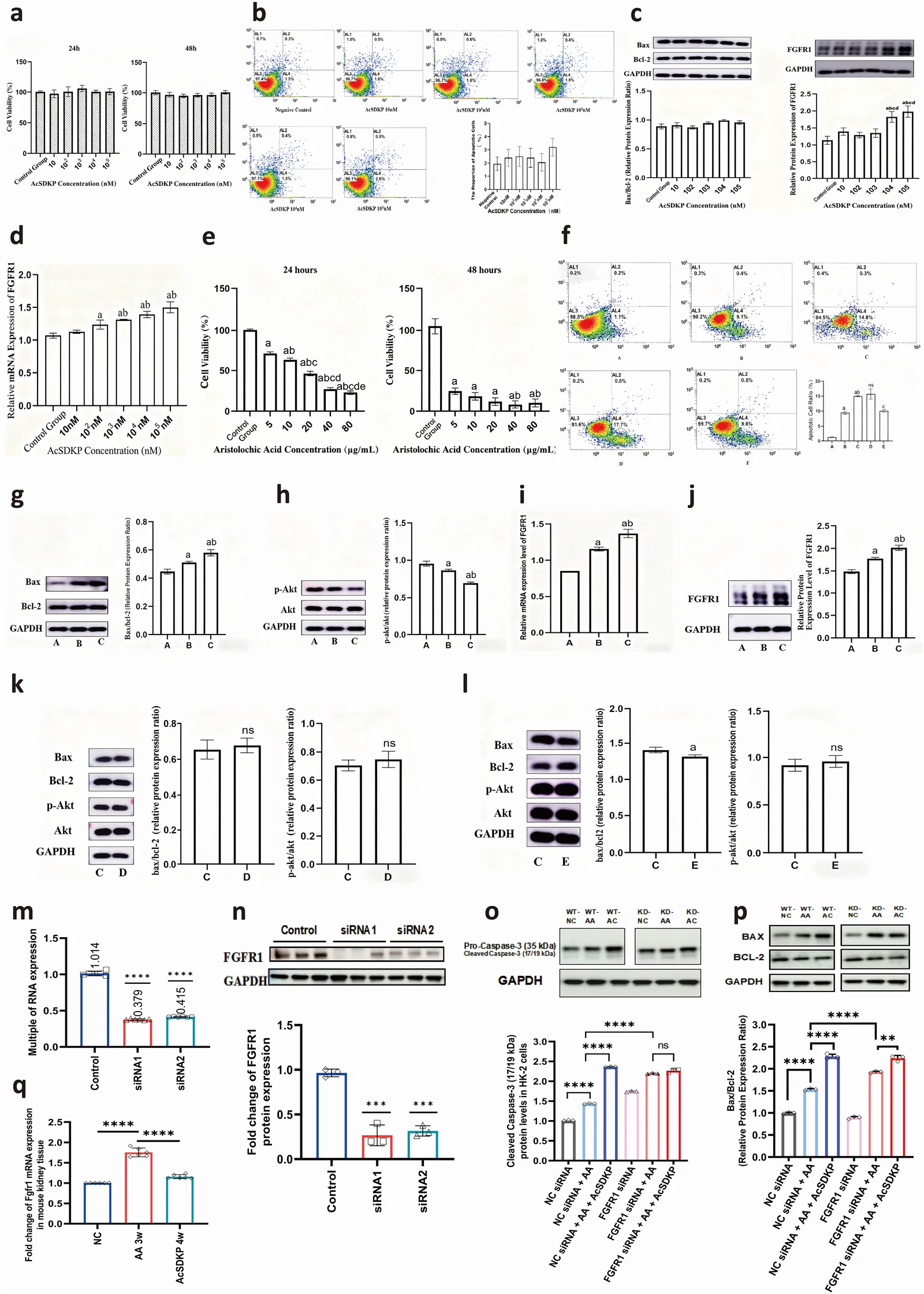
Experiments were repeated three times, and data are presented as mean ± standard deviation. (a) Effects of different concentrations of AcSDKP on HK-2 cell viability after 24 h and 48 h of treatment. CCK-8 assay showed that after HK-2 cells were treated with different concentrations of AcSDKP for 24 h and 48 h, no statistically significant differences in cell viability were observed among groups (*P* > 0.05). (b) Apoptosis status of HK-2 cells treated with different concentrations of AcSDKP for 24 h. No statistically significant differences in HK-2 cell apoptosis were found among groups (*P* > 0.05).(c) Expression of apoptotic proteins and *FGFR1* protein in HK-2 cells treated with different concentrations of AcSDKP for 24 h. (1) No statistically significant differences in the *Bax*/*Bcl-2* ratio of HK-2 cells were observed among groups (*P* > 0.05); (2) For *FGFR1* protein expression: a*P* < 0.05 vs. control group; b*P* < 0.05 vs. 10 nM group; c*P* < 0.05 vs. 10² nM group; d*P* < 0.05 vs. 10³ nM group. (d) *FGFR1* mRNA expression in HK-2 cells treated with different concentrations of AcSDKP for 24 h. a*P* < 0.05 vs. control group; b*P* < 0.05 vs. 10 nM group. (e) AA inhibited HK-2 cell viability in a dose- and time-dependent manner. a*P* < 0.05 vs. control group; b*P* < 0.05 vs. 5 μg/mL group; c*P* < 0.05 vs. 10 μg/mL group; d*P* < 0.05 vs. 20 μg/mL group; d*P* < 0.05 vs. 40 μg/mL group. (f) Apoptosis status of HK-2 cells under AA exposure with different treatments. a*P* < 0.05 vs. Group A; b*P* < 0.05 vs. Group B; ns*P* > 0.05 vs. Group C; a*P* < 0.05 vs. Group C (ns = not significant). (g) Effect of AcSDKP on apoptotic proteins in HK-2 cells under AA exposure. a*P* < 0.05 vs. Group A; b*P* < 0.05 vs. Group B. (h) Effect of AcSDKP on the *Akt* signaling pathway in HK-2 cells under AA exposure. a*P* < 0.05 vs. Group A; b*P* < 0.05 vs. Group B. (i) Effect of AcSDKP on *FGFR1* mRNA expression in HK-2 cells under AA exposure. a*P* < 0.05 vs. Group A; b*P* < 0.05 vs. Group B. (j) Effect of AcSDKP on *FGFR1* protein expression in HK-2 cells under AA exposure. a*P* < 0.05 vs. Group A; b*P* < 0.05 vs. Group B. (k) Effect of *FGFR1* inhibitor PD173074 pretreatment on apoptosis of HK-2 cells under AA exposure. ns*P* > 0.05 vs. Group C. (l) Effect of FGF2 pretreatment on apoptosis of HK-2 cells under AA exposure. ns*P* > 0.05 vs. Group C; a*P* < 0.05 vs. Group C. (m) RT-qPCR analysis of *FGFR1* mRNA expression validating the knockdown efficiency (60.30%) in HK-2 cells transfected with *FGFR1*-specific siRNA compared to negative control (NC) siRNA. (n)Representative Western blot and corresponding densitometric quantification confirming the suppression of *FGFR1* protein levels (70.91%). (o)Representative Western blots and quantitative analysis of *Pro-Caspase-3* (~35 kDa) and its active cleaved fragment (*Cleaved Caspase-3*, 17/19 kDa) in NC (WT) and *FGFR1*-knockdown (KD) HK-2 cells following AA exposure, with or without AcSDKP pretreatment. *GAPDH* served as the loading control. (p)Representative Western blots and densitometric quantification of *Bax* and *Bcl-2* protein expression, alongside the calculated *Bax/Bcl-2* ratio. Knockdown of *FGFR1* significantly exacerbated AA-induced apoptosis, whereas AcSDKP treatment maintained overwhelmingly high apoptotic signaling in *FGFR1*-depleted cells, indicating an *FGFR1*-independent mechanism of toxicity. Data are expressed as mean ± SD (n = 3 independent experiments). Statistical significance was determined by One-way ANOVA followed by Tukey’s post hoc test. * *P* < 0.05, ** *P* < 0.01, *** *P* < 0.001, **** *P* < 0.0001. (q) RT-qPCR validation of FGFR1 mRNA expression in in vivo mouse kidney tissues across different treatment groups. Data are expressed as mean ± SD (n = 6 independent experiments). **** *P* < 0.0001.

The transcriptomic enrichment of the *VEGF/FGFR1* signaling axis identified by RNA-seq was rigorously supported by orthogonal downstream validations. At the ligand level, our ELISA results (Fig 2I) functionally validated this pathway by demonstrating the robust restoration of VEGF protein secretion in AcSDKP-treated pericytes. To directly validate the receptor activation in vivo, we assessed the mRNA expression of *FGFR1* in the mouse kidney tissues via RT-qPCR. Consistent with the transcriptomic data, AcSDKP treatment notably upregulated *FGFR1* mRNA expression in the AA-injured kidneys (Fig 6q). Having validated the in vivo upregulation of *FGFR1*, we next investigated its specific cellular role in the tubular compartment in vitro.

### 3.4. The mechanism by which AcSDKP exacerbates apoptosis is independent of *FGFR1* signaling, whereas *FGFR1* activation (via FGF2) independently alleviates apoptosis

To investigate the biological effects of AcSDKP on HK-2 cells, we utilized the Cell Counting Kit-8 (CCK-8) assay to evaluate the effects of AcSDKP at varying concentrations (0, 10, 10², 10³, 10⁴, and 105 nM) and treatment durations (24 h and 48 h) on cell viability. Relative to the control group (0 nM AcSDKP), treatment of HK-2 cells with AcSDKP at 10, 10², 10³, 10⁴, or 105 nM for 24 h or 48 h did not induce a statistically significant change in cell viability (*P* > 0.05) (Fig 6a). Thus, under normal culture conditions, AcSDKP exerts no time- or concentration-dependent effect on the proliferation of HK-2 cells.

Following treatment of HK-2 cells with AcSDKP at different concentrations (0, 10, 10², 10³, 10⁴, and 105 nM) for 24 h, flow cytometry was performed to assess cell apoptosis. As shown in Fig 6b, 24-h treatment with AcSDKP at the aforementioned concentrations had no statistically significant effect on HK-2 cell apoptosis. Fig 6c further illustrates that there were no obvious changes in the expression levels of Bax and Bcl-2 in HK-2 cells treated with AcSDKP at different concentrations; additionally, the Bax/Bcl-2 ratio exhibited no statistically significant difference compared with the control group (*P* > 0.05).

Following treatment of HK-2 cells with AcSDKP at different concentrations (0, 10, 10², 10³, 10⁴, and 105 nM) for 24 h, cellular proteins and total RNA were extracted. Western blot analysis and qPCR were then performed respectively to detect the expression of *FGFR1*. As shown in Fig 6c, compared with the control group (0 nM AcSDKP), the expression level of FGFR1 in HK-2 cells increased in a concentration-dependent manner with rising AcSDKP concentrations. Specifically, when AcSDKP concentrations reached 10⁴ nM and 105 nM, FGFR1 expression in HK-2 cells was significantly higher than that in the control group (*P* < 0.05). Furthermore, qPCR results demonstrated that the mRNA expression level of *FGFR1* in HK-2 cells also increased as the concentration of AcSDKP increased (Fig 6d).

Under normal cell culture conditions, AcSDKP did not affect the proliferation or apoptosis of HK-2 cells in a time- or concentration-dependent manner. Given that both the protein and mRNA expression levels of *FGFR1* in HK-2 cells were significantly upregulated when treated with 10⁴ nM AcSDKP, this concentration was selected for subsequent investigations into the effect of AcSDKP on HK-2 cell apoptosis under AA-exposed conditions, as well as the underlying mechanism.

The CCK-8 assay was first used to assess the impact of AA on HK-2 cell viability. HK-2 cells were exposed to AA at concentrations of 0, 5, 10, 20, 40, and 80 μg/mL for 24 h and 48 h respectively. As shown in Fig 6e, AA exerted cytotoxic effects on HK-2 cells. Compared with the control group (0 μg/mL AA), AA inhibited HK-2 cell viability in a dose- and time-dependent manner. Based on these results, treatment of HK-2 cells with 10 μg/mL AA for 24 h was used to establish the AA-induced renal tubular epithelial cell injury model in this study.

Flow cytometry revealed the apoptosis rate of HK-2 cells in Group B was significantly higher than that in Group A. The apoptosis rate in Group C was even higher, which was significantly greater than those in both Groups A and B (Fig 6f).

Western blot showed that, compared with Group A, Group B exhibited upregulated expression of Bax and a significantly increased Bax/Bcl-2 ratio (P < 0.05). In Group C, Bax expression was further upregulated, and the increase in the Bax/Bcl-2 ratio was statistically significant (P < 0.05) (Fig 6g).

Previous studies have demonstrated that AA induces cell apoptosis by inhibiting the *Akt* signaling pathway, which is accompanied by upregulation of *Bax* expression and downregulation of *Bcl-2* expression [17]. We also observed that compared with the Group A, treatment with Group B significantly downregulated the expression of phosphorylated Akt (p-Akt) and markedly reduced the p-Akt/Akt ratio (*P* < 0.05). Furthermore, pretreatment with Group C further suppressed the expression of p-Akt in HK-2 cells, leading to a more significant decrease in the p-Akt/Akt ratio (*P* < 0.05) (Fig 6h). Thus, pretreatment with AcSDKP can further inhibit the expression of p-Akt in HK-2 cells under AA-exposed conditions.

Relative to group A, group B significantly upregulated the expression of *FGFR1* in HK-2 cells at both the protein and mRNA levels. Notably, Group C resulted in a further increase in *FGFR1* expression at both the protein and mRNA levels (*P* < 0.05) (Fig 6i, 6j). Collectively, these findings indicate that AA can induce *FGFR1* expression in HK-2 cells, and pretreatment with AcSDKP can further upregulate *FGFR1* expression.

We found that AcSDKP exerts no protective effect on HK-2 cells exposed to AA; instead, it exacerbates apoptosis in these cells. Furthermore, AcSDKP further induces the expression of *FGFR1* and inhibits the *Akt* signaling pathway in HK-2 cells under AA-exposed conditions.

Flow cytometry and Western blot analyses revealed that Group D did not significantly alter the apoptosis rate or the expression of apoptotic proteins (Bax, Bcl-2) and *Akt* pathway-related proteins (p-Akt/Akt ratio) compared to Group C (P > 0.05) (Fig 6k). This indicates that the AcSDKP-induced exacerbation of apoptosis and *Akt* inhibition are not mediated through the *FGFR1* signaling pathway. Conversely, compared with Group C, pretreatment with the Group E significantly decreased the apoptosis rate (Fig 6f) and reversed the apoptotic protein profile, showing downregulated Bax, upregulated Bcl-2, and a significantly decreased Bax/Bcl-2 ratio (*P* < 0.05) (Fig 6l). Meanwhile, the p-Akt/Akt ratio remained unchanged (*P* > 0.05). These findings suggest that pharmacological activation of *FGFR1* alleviates AA-induced apoptosis independently of the *Akt* pathway. Consequently, we hypothesized that the endogenous upregulation of *FGFR1* induced by AcSDKP might represent a compensatory protective mechanism of HK-2 cells in a pathogenic environment, rather than a driver of apoptosis.

### 3.5. Functional Dissection of *FGFR1*: Endogenous *FGFR1* Acts as a Compensatory Survival Factor

To definitively resolve whether the endogenous upregulation of *FGFR1* acts as a driver of injury or a compensatory protector, we performed targeted knockdown of *FGFR1* using small interfering RNA (siRNA) in HK-2 cells. The knockdown efficiency was validated to be 60.30% at the mRNA (Fig 6m) and 70.91% at the protein levels (Fig 6n).

We then evaluated the activation of *Caspase-3*, evidenced by the accumulation of its 17/19 kDa cleaved active fragment from the ~ 35 kDa *Pro-Caspase-3* precursor (Fig 6o), alongside the expression of *Bax* and *Bcl-2* (Fig 6p). In negative control siRNA-transfected cells (WT), AA exposure markedly induced apoptosis, as indicated by a increased Bax/Bcl-2 ratio and Cleaved Caspase-3 levels, which were further exacerbated by AcSDKP treatment. *FGFR1* knockdown alone significantly aggravated AA-induced apoptosis compared to the WT-AA group, resulting in dramatically elevated Cleaved Caspase-3 and Bax levels. This robustly demonstrates that the endogenous upregulation of *FGFR1* is a vital compensatory survival mechanism rather than a pro-apoptotic driver; its removal leaves tubular cells highly vulnerable to AA toxicity.

Furthermore, in *FGFR1*-depleted cells, AcSDKP treatment failed to rescue the cells and maintained overwhelmingly high levels of apoptotic markers (Fig 6o, p). Collectively, these results confirm that AcSDKP’s toxicity in this model is strictly independent of *FGFR1.* The AcSDKP-induced *FGFR1* upregulation is a failed compensatory survival attempt, which is ultimately overpowered by AcSDKP’s concurrent inhibition of the *Akt* pathway.

## 4. Discussion

In the present study, we elucidate a compartment-specific, dual role of AcSDKP in the progression of CKD. On one hand, AcSDKP exerts potent anti-fibrotic and pro-angiogenic effects in the renal interstitium by inhibiting PMT and restoring pericyte-derived VEGF secretion. On the other hand, in the tubular compartment under AA stress, AcSDKP unexpectedly exacerbates epithelial apoptosis. By integrating a self-controlled in vivo model with targeted in vitro gene silencing, we explored the optimal therapeutic window for AcSDKP and dissected the mechanistic paradox surrounding the *VEGF/FGFR1* signaling axis.

Previous studies have established that pericyte detachment and subsequent PMT not only drive ECM deposition but also directly cause PTC rarefaction, leading to hypoxia and accelerated CKD progression [18,19]. Our findings support and extend this paradigm. We demonstrated that TGF-β1-induced PMT drastically impaired the secretory profile of pericytes. Our incorporated ELISA and co-culture data revealed that AcSDKP treatment effectively reprogrammed pericytes to restore *VEGF* secretion, which in turn rescued endothelial CD31 expression, migration, and tubulogenesis. This functional in vitro correlation mirrors our in vivo RNA-seq data, which identified the *VEGF/FGFR1* pathway as the primary signaling axis enriched by early AcSDKP intervention. Collectively, these data confirm that AcSDKP disrupts the vicious cycle of fibrosis and capillary loss by facilitating *VEGF*-mediated pericyte-endothelial crosstalk.

However, the response of the tubular epithelium to AcSDKP presents a complex conundrum. While our data and existing literature acknowledge the generally protective, anti-apoptotic role of *FGFR1* activation in renal tubular cells [20,21], we observed a paradoxical phenomenon: AcSDKP exacerbated AA-induced HK-2 cell apoptosis despite significantly upregulating *FGFR1*. To determine whether *FGFR1* acted as a driver of injury or a bystander, we employed targeted siRNA knockdown. Silencing *FGFR1* dramatically aggravated AA-induced apoptosis, providing functional evidence that the endogenous upregulation of *FGFR1* is a compensatory, protective response attempting to mitigate toxic injury. The failure of AcSDKP to rescue these cells, even in the presence of upregulated *FGFR1*, confirms that its pro-apoptotic toxicity in this specific AA model is fundamentally independent of *FGFR1* signaling.

Instead of acting through *FGFR1*, our signaling data suggest that AcSDKP concurrently inhibits the *Akt* survival pathway in AA-injured tubules, a blockade that ultimately overwhelms the protective capacity of *FGFR1*. Nevertheless, we acknowledge that attributing the cellular demise solely to *Akt* inhibition may be an oversimplification. Because our analysis primarily focused on *Akt* phosphorylation, the potential involvement of parallel or alternative cell death cascades, including *MAPK/ERK* hyperactivation, *NF-κB* inflammatory signaling, and specific programmed necrosis pathways, cannot be entirely excluded. Further comprehensive phosphoproteomic studies are warranted to clarify these mechanisms.

Although the AA model was selected to align the in vivo injury context with our AA-exposed HK-2 model and to examine a toxin-induced CKD setting, we acknowledge that CKD-associated fibrosis is heterogeneous. Prior studies in UUO models have reported anti-fibrotic effects of Ac-SDKP, including reduced extracellular matrix deposition, myofibroblast accumulation, macrophage infiltration, and profibrotic mediator expression [11,22]. Therefore, AcSDKP may also exert anti-fibrotic activity in obstructive nephropathy. However, the tubular stress response observed in our AA model may not fully extrapolate to UUO or renal ischemia-reperfusion injury, where obstruction-driven pressure injury, inflammatory recruitment, hypoxia-reoxygenation, and mitochondrial injury predominate. Future studies using UUO and I/R models are needed to determine whether the VEGF/FGFR1-mediated pericyte-endothelial repair and the FGFR1-independent tubular apoptotic response observed here are conserved across etiologically distinct forms of kidney fibrosis.

From a translational perspective, our self-controlled in vivo model yields two insights. First, regarding therapeutic timing, early AcSDKP intervention (Week 3) successfully halted fibrosis and preserved capillaries, whereas late intervention (Week 4) was largely ineffective. This highlights a stringent therapeutic window, emphasizing the necessity of intervening before PTC rarefaction crosses a “point of no return.” Second, to address concerns that the unilateral self-controlled design might be confounded by systemic hemodynamic shifts, we systematically monitored systemic renal function. The parallel improvement in systemic BUN and SCr in early-treated animals correlates with local histological recovery, confirming that the observed anti-fibrotic effects reflect a genuine structural and functional rescue of the renal parenchyma rather than systemic artifacts. In terms of clinical translation, while our in vivo model highlights the potent early-stage efficacy of AcSDKP, we acknowledge the clinical limitations regarding its application in human CKD progression. Clinically, initiating treatment during the early stages of CKD is inherently challenging, as patients are frequently asymptomatic. However, recent clinical studies, such as the cohort analyzed by Nitta et al., have demonstrated that urinary AcSDKP can serve as a sensitive early biomarker of renal functional decline, even in normoalbuminuric patients with diabetes [23]. This suggests that routine monitoring of such biomarkers could help clinicians identify patients within the optimal early therapeutic window before irreversible capillary rarefaction occurs.

Furthermore, the direct clinical administration of AcSDKP faces severe pharmacokinetic (PK) challenges. As extensively reviewed by Kumar and Yin, AcSDKP is an endogenous tetrapeptide that is rapidly hydrolyzed by the N-terminal active site of angiotensin-converting enzyme (ACE), resulting in an extremely short biological half-life of approximately 4.5 minutes in circulation [24]. Additionally, systemic physiological alterations that occur during CKD progression, including progressive reductions in glomerular filtration and active tubular secretion, further complicate the prediction of drug disposition and clearance [25]. Maintaining a stable, effective dosing regimen via exogenous peptide infusion is therefore clinically impractical.

To overcome these barriers, alternative dosing regimens must be considered. One highly feasible clinical strategy is leveraging ACE inhibitors (ACEi). Because ACE is the primary enzyme responsible for AcSDKP degradation, standard ACEi therapies have been shown to elevate endogenous plasma and tissue AcSDKP levels by 4- to 5-fold, which substantially contributes to their reno-protective effects [24,26]. Translating our findings to human CKD may thus rely on optimizing ACEi-based regimens to maximize endogenous AcSDKP accumulation. Alternatively, the development of degradation-resistant AcSDKP structural analogs or kidney-targeted nanoparticle delivery systems will be essential to sustain local therapeutic concentrations while minimizing the need for continuous systemic infusion.

In summary, AcSDKP functions as a double-edged sword in CKD. Its exceptional ability to halt PMT and promote VEGF-driven endothelial repair makes it a compelling candidate for targeted vascular therapy in fibrotic diseases. However, its complex pro-apoptotic interactions in severely damaged tubular epithelium necessitate cautious, stage-specific application. These mechanistic insights provide a foundation for refining AcSDKP-based therapies in nephrology.

## 5. Conclusion

Our study delineates the double-edged nature of AcSDKP in the pathogenesis of chronic kidney disease. We provide functional evidence that AcSDKP disrupts the vicious cycle of renal fibrosis and microvascular rarefaction by inhibiting pericyte transdifferentiation and reinstating *VEGF*-mediated pericyte-endothelial crosstalk. Our findings offer new insights into the mechanistic paradox surrounding tubular *FGFR1* signaling, suggesting that its upregulation likely serves as a compensatory survival response rather than a primary driver of injury, an effect that appears to be counteracted by AcSDKP-induced *Akt* inhibition. These findings carry significant translational implications. The robust efficacy of early AcSDKP treatment in our self-controlled in vivo model emphasizes a strict, time-sensitive therapeutic window before irreversible capillary dropout occurs. Given its potent vascular benefits but inherent pharmacokinetic challenges (such as rapid ACE-mediated degradation) and potential tubular toxicity under severe stress, the clinical advancement of AcSDKP therapies will likely depend on early disease detection (e.g., using urinary biomarkers) and the development of kidney-targeted delivery systems.

## Supporting information

S1 File Original unadjusted and uncropped images for blots and gels. (7Z)

S2 File The raw sequence data supporting the findings of this study. (ZIP)
