# Supplementary material for: A dual role of AcSDKP in chronic kidney disease: Targeting pericyte-driven fibrosis and vascular repair via VEGF/FGFR1 while eliciting a compensatory FGFR1 upregulation in apoptotic tubular cells
Source: PLoS One. 2026 Sep 11;21(9):e0355409. doi: 10.1371/journal.pone.0355409 (PMC13567759; doi:10.1371/journal.pone.0355409)
Supplement: Supplementary file 1 [file pone.0355409.s004.docx]

**Part I: Response to Reviewer #1**

Reviewer #1 Summary: *Zhu et al., investigate the dual role of AcSDKP in CKD pathogenesis and therapy both in vitro and in vivo models... All of this data collectively links AcSDKP to pericyte-driven fibrosis suppression with endothelial repair, but also reveal potential pro-apoptotic effects in damaged tubular epithelium.*

**Response:** We sincerely thank the reviewer for this excellent and precise summary of our work. We deeply appreciate your recognition of the study's significance and your highly constructive suggestions, which have fundamentally strengthened our mechanistic evidence.

**Major comments**

*1. The FGFR1 data is ambiguous and would benefit from further characterization. While the authors find FGFR1 upregulation in both protective and pro-apoptotic contexts, the data presented in not enough to determine whether FGFR1 acts as a driver or bystander in these phenotypes. The paradox here, which is that AcSDKP upregulates FGFR1 but worsens apoptosis, requires further mechanistic dissection in my opinion. Functional experiments like knockdown or knockout of FGFR1 using shRNA or CRISPR/Cas9 based approaches in HK-2 cells may help address this concern.*

**Response:** We wholeheartedly agree with the reviewer’s brilliant insight. To explicitly address this paradox and determine whether FGFR1 is a driver or bystander, we have performed functional knockdown experiments using FGFR1-specific siRNA in HK-2 cells, as highly recommended. The new results (Figure 6m-p) demonstrate that silencing FGFR1 remarkably exacerbated AA-induced apoptosis (evidenced by sharp increases in Cleaved Caspase-3 and Bax levels). This provides direct functional evidence that the endogenous upregulation of FGFR1 is a compensatory, protective "bystander" attempting to mitigate injury. Crucially, in FGFR1-depleted cells, AcSDKP treatment maintained overwhelmingly high levels of apoptotic markers. This confirms that AcSDKP’s toxicity is strictly FGFR1-independent, likely driven by its concurrent inhibition of the Akt pathway. We have added these new data and thoroughly discussed this mechanism in the revised manuscript. Modifications can be found in the Results section (Page 34, Line 509) and Discussion section (Page 36, Line 560).

*2. The RNA-seq data highlighted in Figure 5 of the VEGF/FGFR1 pathways is compelling but lacks sufficient downstream validation, such as via qPCR or Western blot. Orthogonal quantification of a few key pathway mediators of the highlighted signaling pathways will strengthen this data significantly. Without downstream validation, it is difficult to assess whether this pathway enrichment is functionally relevant or merely associative.*

**Response:** We thank the reviewer for this rigorous methodological suggestion. We completely agree that bulk RNA-seq data require orthogonal validation. To explicitly address this, we have consolidated multi-level downstream validations in the revised manuscript:

**a.Ligand validation (Protein level):** We performed a new VEGF ELISA assay on pericyte supernatants (Figure 2i), confirming that AcSDKP significantly restores VEGF protein secretion.(Page 19, Line 317)

**b.Receptor validation (In vivo mRNA level):** We added new RT-qPCR data from the in vivo mouse kidney tissues (Figure 6q), which directly corroborates the transcriptomic upregulation of FGFR1.(Page 30, Line 437)

**c.Receptor validation (In vitro mRNA/Protein levels):** The target was further quantitatively validated in HK-2 cells via WB and qPCR (Figure 6m, 6n). These combined functional and quantitative data successfully validate the RNA-seq enrichment. Modifications can be found in the Results section (Page 30, Line 437).

*3. The self-controlled unilateral CKD model is novel but introduces confounding factors. Using one kidney as the control and the contralateral as treated ignores systemic effects of AcSDKP, such as altered hemodynamics and circulating cytokines. Parallel groups of sham-operated control animals receiving AcSDKP would strengthen interpretation of the in vivo data.*

**Response:** We fully understand the reviewer's legitimate concern regarding systemic confounding factors. To strictly adhere to the 3R principles of animal welfare (Reduction) while addressing this concern, we systemically monitored Blood Urea Nitrogen (BUN) and serum creatinine (SCr) levels before and after treatment. As shown in our newly added Figure 4h,4i, the parallel and significant reduction in systemic BUN/SCr in the early-intervention group strongly correlates with the local histological recovery in the right kidney. This functional concordance confirms that the observed anti-fibrotic effects reflect a genuine therapeutic rescue of the renal parenchyma rather than mere systemic hemodynamic artifacts. We have expanded on this limitation and its counter-evidence in the revised Discussion. Modifications can be found in the Results section (Page 25, Line 384) and Discussion section (Page 37, Line 581).

*4. There seems to be limited functional correlation between the Pericyte and Endothelial cell data. The authors propose that AcSDKP restores vascular repair through pericyte-mediated VEGF secretion, measuring VEGF levels in pericyte supernatants would be significantly useful here. Similarly, confirming endothelial FGFR1 activation downstream would also help.*

**Response:** We deeply appreciate this excellent suggestion. As suggested, we have quantified VEGF levels in the pericyte culture supernatants using ELISA. The new data (Figure 2i) demonstrate that TGF-β1 impaired the secretory function of pericytes, whereas AcSDKP robustly restored VEGF secretion (*P* < 0.001). This provides the missing functional link, proving that AcSDKP rescues the pericyte-endothelial crosstalk via VEGF paracrine signaling. Modifications can be found in the Results section (Page 19, Line 317).

*5. The authors conclude that AcSDKP-induced apoptosis is “Akt-independent,” yet only p-Akt and total Akt are measured. Evaluation of alternative death pathways would help strengthen this conclusion.*

**Response:** We apologize for the overly absolute wording in our previous draft. We completely agree that relying solely on *p-Akt/Akt* is insufficient to rule out all other pathways. Accordingly, we have softened this conclusion throughout the text. In the revised Discussion, we acknowledge this limitation and propose that while Akt inhibition plays a dominant role in overriding FGFR1 protection, alternative or parallel cell death cascades (such as *MAPK/ERK* hyperactivation or *NF-κB* inflammatory signaling) cannot be completely ruled out and warrant future phosphoproteomic investigation. Modifications can be found in the Discussion section (Page 37, Line 571).

**Minor comments**

1. *Scale bars in most microscopy-based images are either missing or very small. Please add or increase the size of scale bars.*

**Response:** We apologize for this oversight. We have carefully revised all microscopy-based images (especially Figures 3 and 4) to ensure that the scale bars are prominently displayed, clear, and clearly stated in the figure legends.(Page 22, Line 351) (Page 25, Line 384)

2. *Although this study identifies early-stage efficacy of AcSDKP, the clinical feasibility of early intervention in CKD patients is limited. The Discussion section should better contextualize issues such as pharmacokinetic challenges, dosing regimens, and relevance to human CKD progression.*

**Response:** Thank you for pointing out this critical translational aspect. We have significantly expanded the Discussion to address these points. Specifically, we cited recent clinical findings (e.g., Nitta et al., 2019) demonstrating urinary AcSDKP as an early biomarker in human CKD. We also thoroughly discussed the pharmacokinetic challenges of AcSDKP (e.g., rapid degradation by ACE, extremely short half-life) and proposed feasible clinical dosing regimens, such as leveraging ACE inhibitors to elevate endogenous AcSDKP or developing nanoparticle delivery systems. Modifications can be found in the Discussion section (Page 38, Line 594).

3. *The discussion section is lengthy. A tighter focus on the novel mechanistic insights would be useful to readers and enhance impact.*

**Response:** We completely agree. We have entirely rewritten and condensed the Discussion section. Redundant background information has been removed. The revised Discussion is now tightly focused on the dual role of AcSDKP, the FGFR1 paradox (incorporating the new siRNA data), model limitations, and clinical pharmacokinetics, making it much more concise and impactful.

**Part II: Response to Reviewer #2**

*Reviewer #2 Summary: The text is mostly confusing... I request that the authors rewrite the text following the guidelines... A crucial segment is missing: Statistical analysis!... I still couldn’t understand the animal experiment... Finally, the authors used ether as method of euthanasia...*

**Response:** We sincerely appreciate the reviewer’s strict and highly detailed scrutiny of our manuscript. Your rigorous standards have helped us identify and correct several critical flaws in our presentation, methodology description, and ethical documentation. We have implemented a massive overhaul of the manuscript to address every single concern you raised.

*1. Text is confusing and excessively long. Rewrite the text following guidelines, eliminate redundancy. Submit the text to a specialist in the English language.* **Response:** We deeply apologize for the confusing structure and redundant text in the original draft. We have systematically restructured and significantly shortened the Introduction and Discussion sections, eliminating irrelevant background information. The entire manuscript has undergone professional English language polishing to ensure clarity, proper flow, and adherence to journal guidelines.

2. *A crucial segment is missing: Statistical analysis! Which statistical analysis did the authors use? Did the authors use any normality test?*

**Response:** We sincerely apologize for omitting this crucial section. We have now added a comprehensive "Statistical Analysis" subsection in the Materials and Methods. Data are presented as mean ± standard deviation (SD) from at least three independent experiments. Normality of the data distribution was assessed using the Shapiro-Wilk test. For comparisons between two independent groups, an unpaired two-tailed Student’s t-test was performed. For comparisons among three or more groups, a One-way Analysis of Variance (ANOVA) followed by Tukey’s post hoc test was employed. In cases where the data did not pass the normality test, the non-parametric Mann-Whitney U test (for two groups) or Kruskal-Wallis test followed by Dunn's multiple comparisons test (for multiple groups) was utilized. All statistical analyses and graph generation were performed using GraphPad Prism 9.0 software (GraphPad Software, San Diego, CA, USA). A *P*-value < 0.05 was considered statistically significant. Modifications can be found in the Materials and Methods section (Page 14, Line 257).

3. *Bioinformatics data: I couldn’t find anything about that in the Material and methods. Is this database available?*

**Response:** We appreciate this important reminder. We have added a dedicated "Bioinformatics and RNA-Sequencing" section to the Materials and Methods. Furthermore, we have uploaded our raw sequence data to the Genome Sequence Archive (GSA) at the National Genomics Data Center. The accession number (CRA038202) and a statement of data availability have been prominently included. Modifications can be found in the Materials and Methods section (Page 13, Line 245).

4. *Regarding antibodies used: missing companies, catalog numbers, and concentrations.*

**Response:** We have meticulously updated the "Western Blot Analysis" and "Histology and Immunofluorescence" sections. All primary and secondary antibodies are now listed with their respective providing companies, specific catalog numbers, and working dilution concentrations. Modifications can be found in the Materials and Methods section (Page 11, Line 211).

5. *RT-qPCR primers not mentioned. Disclose the primer sequence. Gene names should be in italics.*

**Response:** Thank you for the correction. We have now provided all primer sequences in a newly added **Table 1** and specified that they were commercially synthesized. Furthermore, we have carefully reviewed the entire manuscript and formatted all gene/mRNA names in italics (e.g., *FGFR1, Hif1a*) following standard nomenclature guidelines. Modifications can be found in **Table 1** and throughout the manuscript.(Page 12, Line 232)

6. *Animal experiment confusing (3 weeks, 4 weeks, before and after...). Was there any surgery involved? Anesthesia? Biopsy?*

**Response:** We apologize for the previous confusing description. We have completely rewritten the "Animal Models and Experimental Design" section. To ensure absolute clarity, we separated the animal studies into two explicitly titled sub-models: 1) Time-Course Progression Model and 2) Self-Controlled Unilateral Intervention Model. We have clearly detailed that a complete "left nephrectomy was surgically performed" under anesthesia at specific timelines, rather than a biopsy, serving as the pre-treatment baseline. Modifications can be found in the Materials and Methods section (Page 6, Line 111). Flowchart of the experimental procedure for constructing a mouse aristolochic acid nephropathy model can be found in **Figure 3a.** (Figure 3a, Page 22, Line 351)

7. *Euthanasia method: Ether is inhumane and alters physiological parameters. Disclose the animal protocol.*

**Response:** We are extremely grateful for your vigilance regarding animal welfare. We deeply apologize for the documentation error in the original manuscript. The term "ether" was an inadvertent translation/typing error. The actual ethically approved protocol (Approval No. SUDA20240326A04) strictly utilized sodium pentobarbital. In the revised manuscript, we have corrected this and precisely detailed our ethically compliant procedures: Survival blood collection was performed via the tail vein, and terminal blood collection was performed via retro-orbital bleeding under deep sodium pentobarbital anesthesia, followed by cervical dislocation to confirm death, strictly adhering to AVMA and ARRIVE guidelines. Modifications can be found in the Materials and Methods section (Page 6, Line 111).

8. *Figures: resolution, alignment, scale bars, sample size (n). The mention of a "trend" in Hif1a with n=3 is not fair.*

**Response:** We sincerely appreciate your rigorous statistical critique. We fully agree that claiming a "trend" with a sample size of n=3 is scientifically inappropriate. To definitively address this concern and ensure the absolute reliability of our data, we have completely repeated the RT-qPCR experiment for Hif1a with an expanded sample size of n=6. The updated, appropriately powered results have now been incorporated into the revised figure. The results are now described strictly based on the robust statistical findings of the n=6 cohort.Additionally, we have meticulously revised all figures to ensure optimal resolution, proper alignment, and the inclusion of clear scale bars. The precise sample size is now explicitly stated in every figure legend.Modifications can be found in the Results section (Page 19, Line 317) and the corresponding Figure legend.(Page 46, Line 764)

*9. References: Lack proper reference (sub-Saharan Africa, scleroderma, lines 78, 82, 88, 95-99). Doubled refs, unavailable refs, unrelated refs (line 116, 132, 136).* **Response:** We deeply appreciate your rigorous fact-checking and sincerely apologize for the inaccuracies and poor curation in the original reference list. Through a comprehensive rewrite of the Introduction and Discussion sections, we have permanently removed the unsupported statements regarding sub-Saharan Africa, scleroderma models, and all loosely related or inaccurate references (including those originally in lines 116, 132, and 136).

Furthermore, we have significantly elevated the quality and relevance of the literature cited in our revised Discussion. To robustly contextualize our new mechanistic and translational insights, we have integrated several highly relevant, peer-reviewed studies. For instance, we incorporated recent literature detailing the pharmacokinetic challenges of AcSDKP (such as its rapid degradation by ACE and short half-life) and clinical cohort studies highlighting the potential of AcSDKP as an early biomarker in human CKD progression.

Finally, we have thoroughly audited the entire revised Reference list to eliminate any doubled or unavailable citations, ensuring that every reference is strictly accurate, directly pertinent to our findings, and properly formatted according to the journal's guidelines. Modifications can be found throughout the Introduction(Page 4, Line 66), Discussion(Page 35, Line 533), and References(Page 41, Line 666) sections.
