# Supplementary material for: A dual role of AcSDKP in chronic kidney disease: Targeting pericyte-driven fibrosis and vascular repair via VEGF/FGFR1 while eliciting a compensatory FGFR1 upregulation in apoptotic tubular cells
Source: PLoS One. 2026 Sep 11;21(9):e0355409. doi: 10.1371/journal.pone.0355409 (PMC13567759; doi:10.1371/journal.pone.0355409)
Supplement: Supplementary file 2 [file pone.0355409.s005.docx]

Manuscript ID: PONE-D-25-54890R1

Title: A Dual Role of AcSDKP in Chronic Kidney Disease: Targeting Pericyte-Driven Fibrosis and Vascular Repair via VEGF/FGFR1 While Eliciting a Compensatory FGFR1 Upregulation in Apoptotic Tubular Cells

Journal: *PLOS ONE*

Dear Academic Editor and Reviewers,

We sincerely thank the Academic Editor and both reviewers for their constructive evaluation of our revised manuscript. We are grateful that both reviewers considered the previous concerns to have been addressed. In this minor revision, we have focused on the remaining issues raised by Reviewer #2. Specifically, we have added a dedicated disclosure statement on the use of artificial intelligence tools, in accordance with PLOS policy; carefully rechecked the manuscript language and removed unnecessary long-dash punctuation and other stylistic symbols that could distract readers and expanded the Discussion to acknowledge the need to validate AcSDKP in additional kidney fibrosis models, particularly unilateral ureteral obstruction (UUO) and renal ischemia-reperfusion injury (I/R).

All revisions have been incorporated into the marked manuscript and the clean revised manuscript.

# Reviewer #1

**Comment:** All comments have been addressed.

**Response:** We sincerely thank the reviewer for the positive evaluation and for recognizing our efforts to address the previous concerns.

# Reviewer #2

**Comment 1:** I appreciate the authors’ response to the concerns raised by both reviewers. The text is much more comprehensible now. I noticed that the text contains some symbols, like the long dash, that are very characteristic of LLM software. Was any kind of generative AI used for the production of the manuscript? If so, it is important that this is disclosed, including the extension of the use of it (was it for writing the text? Translating it? Reviewing grammar?)

**Response:** We thank the reviewer for raising this important point. We agree that transparency regarding the use of artificial intelligence tools is essential. During preparation of the revised manuscript, Grammarly (Grammarly, Inc.) was used only to assist with English-language polishing, grammar checking, and improvement of sentence clarity. No artificial intelligence tool was used to generate experimental data, analyze data, perform statistical analysis, create figures or images, select or fabricate references, or formulate the scientific hypotheses, interpretations, conclusions, or limitations of the study. All AI-assisted edits were manually checked and approved by the authors against the original experimental data and source materials. The authors take full responsibility for the accuracy, integrity, and originality of all manuscript content.

To address the reviewer’s concern about style, we carefully revised the manuscript language and replaced unnecessary long dashes and AI-like punctuation with standard scientific English punctuation, while retaining only conventional symbols required for scientific notation, ranges, and figure references. We have also added the following dedicated disclosure statement to the Materials and Methods section of the manuscript:

*During the preparation of the manuscript, Grammarly (Grammarly, Inc.) was used solely to assist with English-language polishing, grammar checking, and improvement of sentence clarity. The tool was not used to generate experimental data, analyze data, perform statistical analyses, create figures or images, select references, or develop the scientific hypotheses, interpretations, conclusions, or limitations of the study. All AI-assisted language edits were manually reviewed and verified by the authors against the original experimental records and source materials. The authors take full responsibility for the accuracy, integrity, and originality of the final manuscript content.*

**Comment 2:** I understood the authors chose the aristolochic acid model for it to be replicable to the in vitro models. I wonder how AcSDKP would behave in another model of kidney fibrosis, such as unilateral ureter obstruction or renal ischemia-reperfusion model.

**Response:** We appreciate this insightful suggestion. We selected the aristolochic acid (AA)-induced CKD model because it allows direct alignment between the in vivo injury context and the AA-exposed HK-2 tubular epithelial cell model used in our mechanistic experiments. This design helped us interpret the compartment-specific effects of AcSDKP on pericyte-endothelial crosstalk and tubular epithelial injury within a consistent toxin-induced CKD framework.

We agree, however, that CKD-related fibrosis is etiologically heterogeneous and that additional models are needed to determine whether our findings are generalizable. Previous studies using UUO models have reported anti-fibrotic effects of Ac-SDKP, including reduced extracellular matrix deposition, myofibroblast accumulation, macrophage infiltration, and profibrotic mediator expression. Therefore, we would expect AcSDKP to retain at least part of its anti-fibrotic and vascular-protective activity in obstructive fibrosis. Nevertheless, the tubular epithelial response in UUO or I/R may differ from that observed in AA nephropathy because these models involve distinct drivers, including obstruction-associated pressure injury, inflammatory recruitment, hypoxia-reoxygenation injury, mitochondrial dysfunction, and AKI-to-CKD transition mechanisms. Thus, the extent to which VEGF/FGFR1-mediated pericyte-endothelial repair and FGFR1-independent tubular apoptosis are conserved across models remains an important question for future investigation.

Accordingly, we have added a limitation and future-direction statement to the Discussion as follows:

*Although the AA model was selected to align the in vivo injury context with our AA-exposed HK-2 model and to examine a toxin-induced CKD setting, we acknowledge that CKD-associated fibrosis is heterogeneous. Prior studies in UUO models have reported anti-fibrotic effects of Ac-SDKP, including reduced extracellular matrix deposition, myofibroblast accumulation, macrophage infiltration, and profibrotic mediator expression. Therefore, AcSDKP may also exert anti-fibrotic activity in obstructive nephropathy. However, the tubular stress response observed in our AA model may not fully extrapolate to UUO or renal ischemia-reperfusion injury, where obstruction-driven pressure injury, inflammatory recruitment, hypoxia-reoxygenation, and mitochondrial injury predominate. Future studies using UUO and I/R models are needed to determine whether the VEGF/FGFR1-mediated pericyte-endothelial repair and the FGFR1-independent tubular apoptotic response observed here are conserved across etiologically distinct forms of kidney fibrosis. (Line 573-586)*

We thank the Academic Editor and the reviewers again for their constructive comments, which have helped us improve the transparency, clarity, and translational framing of the manuscript.

Best regards,

The Authors

Pediatric Clinical Research Institute of Soochow University, Pediatric Clinical Medical College of Soochow University

Department of Nephrology and Immunology, Children’s Hospital of Soochow University, Soochow
