# Supplementary material for: A dual role of AcSDKP in chronic kidney disease: Targeting pericyte-driven fibrosis and vascular repair via VEGF/FGFR1 while eliciting a compensatory FGFR1 upregulation in apoptotic tubular cells
Source: PLoS One. 2026 Sep 11;21(9):e0355409. doi: 10.1371/journal.pone.0355409 (PMC13567759; doi:10.1371/journal.pone.0355409)
Supplement: Peer review history

# Peer review: Decision Letter 0

**DOI:** 10.1371/journal.pone.0355409.r001

**Authors:** Partha Mukhopadhyay

11 Dec 2025

Dear Dr. Li,

plosone@plos.org. When you're ready to submit your revision, log on to https://www.editorialmanager.com/pone/ and select the 'Submissions Needing Revision' folder to locate your manuscript file.

- A rebuttal letter that responds to each point raised by the academic editor and reviewer(s). You should upload this letter as a separate file labeled 'Response to Reviewers'.
- A marked-up copy of your manuscript that highlights changes made to the original version. You should upload this as a separate file labeled 'Revised Manuscript with Track Changes'.
- An unmarked version of your revised paper without tracked changes. You should upload this as a separate file labeled 'Manuscript'.

If you would like to make changes to your financial disclosure, please include your updated statement in your cover letter. Guidelines for resubmitting your figure files are available below the reviewer comments at the end of this letter.

If applicable, we recommend that you deposit your laboratory protocols in protocols.io to enhance the reproducibility of your results. Protocols.io assigns your protocol its own identifier (DOI) so that it can be cited independently in the future. For instructions see: https://journals.plos.org/plosone/s/submission-guidelines#loc-laboratory-protocols. Additionally, PLOS ONE offers an option for publishing peer-reviewed Lab Protocol articles, which describe protocols hosted on protocols.io. Read more information on sharing protocols at https://plos.org/protocols?utm_medium=editorial-email&utm_source=authorletters&utm_campaign=protocols.

We look forward to receiving your revised manuscript.

Kind regards,

Partha Mukhopadhyay, Ph.D.

Section Editor

PLOS One

Journal Requirements:

When submitting your revision, we need you to address these additional requirements.

1.Please ensure that your manuscript meets PLOS ONE's style requirements, including those for file naming. The PLOS ONE style templates can be found at

https://journals.plos.org/plosone/s/file?id=wjVg/PLOSOne_formatting_sample_main_body.pdf and

https://journals.plos.org/plosone/s/file?id=ba62/PLOSOne_formatting_sample_title_authors_affiliations.pdf

2. To comply with PLOS One submissions requirements, in your Methods section, please provide additional information regarding the experiments involving animals and ensure you have included details on (1) methods of sacrifice, (2) methods of anesthesia and/or analgesia, and (3) efforts to alleviate suffering.

3. Please note that funding information should not appear in any section or other areas of your manuscript. We will only publish funding information present in the Funding Statement section of the online submission form. Please remove any funding-related text from the manuscript.

4. Thank you for stating the following financial disclosure:

“This research was supported by grants from the Soochow Key Discipline Project of Pediatric Immunology (grant number SZXK202106) and the Soochow Science and Technology Development Plan Project (grant number SS202067).”

Please state what role the funders took in the study.  If the funders had no role, please state: "The funders had no role in study design, data collection and analysis, decision to publish, or preparation of the manuscript."

If this statement is not correct you must amend it as needed.

Please include this amended Role of Funder statement in your cover letter; we will change the online submission form on your behalf.

5. We note that you have indicated that there are restrictions to data sharing for this study. PLOS only allows data to be available upon request if there are legal or ethical restrictions on sharing data publicly. For more information on unacceptable data access restrictions, please see http://journals.plos.org/plosone/s/data-availability#loc-unacceptable-data-access-restrictions.

Before we proceed with your manuscript, please address the following prompts:

a) If there are ethical or legal restrictions on sharing a de-identified data set, please explain them in detail (e.g., data contain potentially identifying or sensitive patient information, data are owned by a third-party organization, etc.) and who has imposed them (e.g., a Research Ethics Committee or Institutional Review Board, etc.). Please also provide contact information for a data access committee, ethics committee, or other institutional body to which data requests may be sent.

b) If there are no restrictions, please upload the minimal anonymized data set necessary to replicate your study findings to a stable, public repository and provide us with the relevant URLs, DOIs, or accession numbers. For a list of recommended repositories, please see

https://journals.plos.org/plosone/s/recommended-repositories. You also have the option of uploading the data as Supporting Information files, but we would recommend depositing data directly to a data repository if possible.

We will update your Data Availability statement on your behalf to reflect the information you provide.

6. Please amend either the abstract on the online submission form (via Edit Submission) or the abstract in the manuscript so that they are identical.

7. Please include captions for your Supporting Information files at the end of your manuscript, and update any in-text citations to match accordingly. Please see our Supporting Information guidelines for more information: http://journals.plos.org/plosone/s/supporting-information.

8. We are unable to open your Supporting Information file “Figures minimum.7z”. Please kindly revise as necessary and re-upload.

9. PLOS ONE now requires that authors provide the original uncropped and unadjusted images underlying all blot or gel results reported in a submission’s figures or Supporting Information files. This policy and the journal’s other requirements for blot/gel reporting and figure preparation are described in detail at https://journals.plos.org/plosone/s/figures#loc-blot-and-gel-reporting-requirements and https://journals.plos.org/plosone/s/figures#loc-preparing-figures-from-image-files. When you submit your revised manuscript, please ensure that your figures adhere fully to these guidelines and provide the original underlying images for all blot or gel data reported in your submission. See the following link for instructions on providing the original image data: https://journals.plos.org/plosone/s/figures#loc-original-images-for-blots-and-gels.

In your cover letter, please note whether your blot/gel image data are in Supporting Information or posted at a public data repository, provide the repository URL if relevant, and provide specific details as to which raw blot/gel images, if any, are not available. Email us at plosone@plos.org if you have any questions.

10. If the reviewer comments include a recommendation to cite specific previously published works, please review and evaluate these publications to determine whether they are relevant and should be cited. There is no requirement to cite these works unless the editor has indicated otherwise.

[Note: HTML markup is below. Please do not edit.]

Reviewers' comments:

Reviewer's Responses to Questions

**Comments to the Author**

1. Is the manuscript technically sound, and do the data support the conclusions?

Reviewer #1: Partly

Reviewer #2: Partly

**********

2. Has the statistical analysis been performed appropriately and rigorously?

Reviewer #1: Yes

Reviewer #2: I Don't Know

**********

3. Have the authors made all data underlying the findings in their manuscript fully available?

The PLOS Data policy

Reviewer #1: Yes

Reviewer #2: No

**********

4. Is the manuscript presented in an intelligible fashion and written in standard English?

Reviewer #1: Yes

Reviewer #2: No

**********

Reviewer #1: Summary

Zhu et al., investigate the dual role of AcSDKP in CKD pathogenesis and therapy both in vitro and in vivo models. The authors show that AcSDKP suppresses PMT by restoring PDGFRβ and reducing fibrotic and inflammatory markers, thereby mitigating renal fibrosis. In conditioned co-cultures, AcSDKP-treated pericytes enhance endothelial repair through upregulation of CD31 and VEGF, restoring tubulogenesis and migration capacity. In a CKD mouse model, early AcSDKP treatment reduces collagen deposition and preserves capillary integrity, while delayed treatment dampens this phenotype. RNA-Seq analysis reveals enrichment of the VEGF/FGFR1 pathway in AcSDKP-treated kidneys, suggesting a signaling axis that underlies both anti-fibrotic and pro-angiogenic effects. However, in HK-2 tubular epithelial cells, AcSDKP upregulates FGFR1 but also enhances aristolochic acid–induced apoptosis through an Akt-independent mechanism, while FGF2-mediated FGFR1 activation rescues cells from apoptosis. All of this data collectively links AcSDKP to pericyte-driven fibrosis suppression with endothelial repair, but also reveal potential pro-apoptotic effects in damaged tubular epithelium.

Major comments

1. The FGFR1 data is ambiguous and would benefit from further characterization. While the authors find FGFR1 upregulation in both protective and pro-apoptotic contexts, the data presented in not enough to determine whether FGFR1 acts as a driver or bystander in these phenotypes. The paradox here, which is that AcSDKP upregulates FGFR1 but worsens apoptosis, requires further mechanistic dissection in my opinion. Functional experiments like knockdown or knockout of FGFR1 using shRNA or CRISPR/Cas9 based approaches in HK-2 cells may help address this concern.

2. The RNA-seq data highlighted in Figure 5 of the VEGF/FGFR1 pathways is compelling but lacks sufficient downstream validation, such as via qPCR or Western blot. Orthogonal quantification of a few key pathway mediators of the highlighted signaling pathways will strengthen this data significantly. Without downstream validation, it is difficult to assess whether this pathway enrichment is functionally relevant or merely associative.

3. The self-controlled unilateral CKD model is novel but introduces confounding factors. Using one kidney as the control and the contralateral as treated ignores systemic effects of AcSDKP, such as altered hemodynamics and circulating cytokines. Parallel groups of sham-operated control animals receiving AcSDKP would strengthen interpretation of the in vivo data.

4. There seems to be limited functional correlation between the Pericyte and Endothelial cell data. The authors propose that AcSDKP restores vascular repair through pericyte-mediated VEGF secretion, measuring VEGF levels in pericyte supernatants would be significantly useful here. Similarly, confirming endothelial FGFR1 activation downstream would also help.

5. The authors conclude that AcSDKP-induced apoptosis is “Akt-independent,” yet only p-Akt and total Akt are measured. Evaluation of alternative death pathways would help strengthen this conclusion.

Minor comments

1. Scale bars in most microscopy-based images are either missing or very small. Please add or increase the size of scale bars.

2. Although this study identifies early-stage efficacy of AcSDKP, the clinical feasibility of early intervention in CKD patients is limited. The Discussion section should better contextualize issues such as pharmacokinetic challenges, dosing regimens, and relevance to human CKD progression.

3. The discussion section is lengthy. A tighter focus on the novel mechanistic insights would be useful to readers and enhance impact.

Reviewer #2: The authors investigated the use of N-acetyl-seryl-aspartyl-lysyl-proline (AcSDKP) in renal interstitial fibrosis, specifically in process of myofibroblast formation, from pericyte cells. The authors used plenty of techniques, in vitro, in vivo and bioinformatics to defend their thesis of protective function of AcSDKP. Nevertheless, the manuscript shows some issues that need to be addressed.

The text is mostly confusing. Some paragraphs are well written, but others are very hard to follow. I couldn’t understand the animal experiments, although it is described twice in the text. I had trouble checking the technical merit of the paper for being upset with the text. Which is a shame, for it seems, at least roughly, that the data is good. Also, the text seems excessively long. Although this journal has no limit for the text, authors guidelines explicitly (https://journals.plos.org/plosone/s/submission-guidelines) asks the author to be concise. So, I request that the authors rewrite the text following the guidelines, which includes the order of the sections (this journal requests Material and Methods to come before Results, and the Figure captions to be cited in the text, after the first paragraph that cites them), and eliminating redundancy. Also, submit the text to a specialist in the English language for better comprehension.

An important feature of a scientific paper is that its reproducibility, and Material and Methods section is crucial for that. A crucial segment is missing, and this is inadmissible: Statistical analysis! Which statistical analysis did the authors use? Did the authors use any normality test to validate if the analysis is appropriate?

The manuscript shows some bioinformatics data, but I couldn’t find anything about that in the Material and methods section. Did this set of data generate any database? Is this database available? If not, it would be good if it was.

Regarding antibodies used for immunostaining and Western blot, which were the companies that provided those, what was the catalog number, and which was the concentration used for each of them, and each situation.

Now about RT-qPCR, the authors don’t mention which primers were used? Were they commercially available? If so, which company and which catalog number. Were the primers self-designed? If so, you need to disclose the primer sequence. In time, whenever mentioning gene names, they should be written in italics and following the nomenclature of the database available in the authors guidelines webpage (https://journals.plos.org/plosone/s/submission-guidelines), accordingly to rodent or humans, whenever it is shown in the text.

I still couldn’t understand the animal experiment the authors performed. The 3 weeks, 4 weeks, before and after… none of that is clear. Was there any surgery involved? If so, how was it performed? Did it require any anesthesia? Was it biopsy? Please, let it very clear.

Finally, in line 528, the authors used ether as method of euthanasia. Besides it is inhumane, it is not recommend for euthanasia by the AVMA Guidelines for Euthanasia of Animals (https://www.in.gov/boah/files/AVMA_Euthanasia_Guidelines.pdf, page 22), cited by the ARRIVE guidelines the authors claim to follow. Also, ether inhaling could cause changes in some physiological parameters that might or might interfere in the results. Could the authors please disclose the animal protocol that was approved as SUDA20240326A04?

For figures, make sure the microscopic images are in their best resolution for the size, and that they are aligned. Specifically figures 3 and 4. Make sure the magnification and/or scale bars in all figures with microscopy images. Also include sample size (n) for all the experiments. In Results section, lines 209-211, the authors mention a trend in the result of Hif1a, even when in figure legends they cite n=3. With such a small sample size for RT-qPCR, I think it is not fair to say that there’s any trend.

Finally, the authors should pay attention and double-check the references. Some information, especially in the Introduction section, lacks a proper reference. In line 67, it brings some data on sub-Saharan Africa but there’s no reference for such data. Other statements that lack proper reference are in lines: 78 (there’s no reference of scleroderma fibrosis models), 82, 88, 95-99 (quite poorly written paragraph, by the way), 144. Some references are doubled (as 38 is the same as 24, 40 is 11, and 51 is 50), some are not available (25, 53, and 54), or some seem not related to the affirmation they’re supposed to reference: line 116, reference 22, it doesn’t mention MCP-1; line 132, reference 27; line 136, reference 28.

I suppose that after a new version of the text, with a better comprehension of the animal experiments, I could review the technical merits of this segment properly, and the manuscript can get more concise and understandable.

**********

what does this mean?). If published, this will include your full peer review and any attached files.

If you choose “no”, your identity will remain anonymous but your review may still be made public.

**Do you want your identity to be public for this peer review?**  For information about this choice, including consent withdrawal, please see our Privacy Policy

Reviewer #1: No

Reviewer #2: No

**********

[NOTE: If reviewer comments were submitted as an attachment file, they will be attached to this email and accessible via the submission site. Please log into your account, locate the manuscript record, and check for the action link "View Attachments". If this link does not appear, there are no attachment files.]

To ensure your figures meet our technical requirements, please review our figure guidelines: https://journals.plos.org/plosone/s/figures

You may also use PLOS’s free figure tool, NAAS, to help you prepare publication quality figures: https://journals.plos.org/plosone/s/figures#loc-tools-for-figure-preparation.

NAAS will assess whether your figures meet our technical requirements by comparing each figure against our figure specifications.

# Author comment: Author response to Decision Letter 1

**DOI:** 10.1371/journal.pone.0355409.r002

4 Mar 2026

Part I: Response to Reviewer #1

Reviewer #1 Summary: Zhu et al., investigate the dual role of AcSDKP in CKD pathogenesis and therapy both in vitro and in vivo models... All of this data collectively links AcSDKP to pericyte-driven fibrosis suppression with endothelial repair, but also reveal potential pro-apoptotic effects in damaged tubular epithelium.

Response: We sincerely thank the reviewer for this excellent and precise summary of our work. We deeply appreciate your recognition of the study's significance and your highly constructive suggestions, which have fundamentally strengthened our mechanistic evidence.

Major comments

1. The FGFR1 data is ambiguous and would benefit from further characterization. While the authors find FGFR1 upregulation in both protective and pro-apoptotic contexts, the data presented in not enough to determine whether FGFR1 acts as a driver or bystander in these phenotypes. The paradox here, which is that AcSDKP upregulates FGFR1 but worsens apoptosis, requires further mechanistic dissection in my opinion. Functional experiments like knockdown or knockout of FGFR1 using shRNA or CRISPR/Cas9 based approaches in HK-2 cells may help address this concern.

Response: We wholeheartedly agree with the reviewer’s brilliant insight. To explicitly address this paradox and determine whether FGFR1 is a driver or bystander, we have performed functional knockdown experiments using FGFR1-specific siRNA in HK-2 cells, as highly recommended. The new results (Figure 6m-p) demonstrate that silencing FGFR1 remarkably exacerbated AA-induced apoptosis (evidenced by sharp increases in Cleaved Caspase-3 and Bax levels). This provides direct functional evidence that the endogenous upregulation of FGFR1 is a compensatory, protective "bystander" attempting to mitigate injury. Crucially, in FGFR1-depleted cells, AcSDKP treatment maintained overwhelmingly high levels of apoptotic markers. This confirms that AcSDKP’s toxicity is strictly FGFR1-independent, likely driven by its concurrent inhibition of the Akt pathway. We have added these new data and thoroughly discussed this mechanism in the revised manuscript. Modifications can be found in the Results section (Page 34, Line 509) and Discussion section (Page 36, Line 560).

2. The RNA-seq data highlighted in Figure 5 of the VEGF/FGFR1 pathways is compelling but lacks sufficient downstream validation, such as via qPCR or Western blot. Orthogonal quantification of a few key pathway mediators of the highlighted signaling pathways will strengthen this data significantly. Without downstream validation, it is difficult to assess whether this pathway enrichment is functionally relevant or merely associative.

Response: We thank the reviewer for this rigorous methodological suggestion. We completely agree that bulk RNA-seq data require orthogonal validation. To explicitly address this, we have consolidated multi-level downstream validations in the revised manuscript:

a.Ligand validation (Protein level): We performed a new VEGF ELISA assay on pericyte supernatants (Figure 2i), confirming that AcSDKP significantly restores VEGF protein secretion.(Page 19, Line 317)

b.Receptor validation (In vivo mRNA level): We added new RT-qPCR data from the in vivo mouse kidney tissues (Figure 6q), which directly corroborates the transcriptomic upregulation of FGFR1.(Page 30, Line 437)

c.Receptor validation (In vitro mRNA/Protein levels): The target was further quantitatively validated in HK-2 cells via WB and qPCR (Figure 6m, 6n). These combined functional and quantitative data successfully validate the RNA-seq enrichment. Modifications can be found in the Results section (Page 30, Line 437).

3. The self-controlled unilateral CKD model is novel but introduces confounding factors. Using one kidney as the control and the contralateral as treated ignores systemic effects of AcSDKP, such as altered hemodynamics and circulating cytokines. Parallel groups of sham-operated control animals receiving AcSDKP would strengthen interpretation of the in vivo data.

Response: We fully understand the reviewer's legitimate concern regarding systemic confounding factors. To strictly adhere to the 3R principles of animal welfare (Reduction) while addressing this concern, we systemically monitored Blood Urea Nitrogen (BUN) and serum creatinine (SCr) levels before and after treatment. As shown in our newly added Figure 4h,4i, the parallel and significant reduction in systemic BUN/SCr in the early-intervention group strongly correlates with the local histological recovery in the right kidney. This functional concordance confirms that the observed anti-fibrotic effects reflect a genuine therapeutic rescue of the renal parenchyma rather than mere systemic hemodynamic artifacts. We have expanded on this limitation and its counter-evidence in the revised Discussion. Modifications can be found in the Results section (Page 25, Line 384) and Discussion section (Page 37, Line 581).

4. There seems to be limited functional correlation between the Pericyte and Endothelial cell data. The authors propose that AcSDKP restores vascular repair through pericyte-mediated VEGF secretion, measuring VEGF levels in pericyte supernatants would be significantly useful here. Similarly, confirming endothelial FGFR1 activation downstream would also help.

Response: We deeply appreciate this excellent suggestion. As suggested, we have quantified VEGF levels in the pericyte culture supernatants using ELISA. The new data (Figure 2i) demonstrate that TGF-β1 impaired the secretory function of pericytes, whereas AcSDKP robustly restored VEGF secretion (P < 0.001). This provides the missing functional link, proving that AcSDKP rescues the pericyte-endothelial crosstalk via VEGF paracrine signaling. Modifications can be found in the Results section (Page 19, Line 317).

5. The authors conclude that AcSDKP-induced apoptosis is “Akt-independent,” yet only p-Akt and total Akt are measured. Evaluation of alternative death pathways would help strengthen this conclusion.

Response: We apologize for the overly absolute wording in our previous draft. We completely agree that relying solely on p-Akt/Akt is insufficient to rule out all other pathways. Accordingly, we have softened this conclusion throughout the text. In the revised Discussion, we acknowledge this limitation and propose that while Akt inhibition plays a dominant role in overriding FGFR1 protection, alternative or parallel cell death cascades (such as MAPK/ERK hyperactivation or NF-κB inflammatory signaling) cannot be completely ruled out and warrant future phosphoproteomic investigation. Modifications can be found in the Discussion section (Page 37, Line 571).

Minor comments

1.Scale bars in most microscopy-based images are either missing or very small. Please add or increase the size of scale bars.

Response: We apologize for this oversight. We have carefully revised all microscopy-based images (especially Figures 3 and 4) to ensure that the scale bars are prominently displayed, clear, and clearly stated in the figure legends.(Page 22, Line 351) (Page 25, Line 384)

2.Although this study identifies early-stage efficacy of AcSDKP, the clinical feasibility of early intervention in CKD patients is limited. The Discussion section should better contextualize issues such as pharmacokinetic challenges, dosing regimens, and relevance to human CKD progression.

Response: Thank you for pointing out this critical translational aspect. We have significantly expanded the Discussion to address these points. Specifically, we cited recent clinical findings (e.g., Nitta et al., 2019) demonstrating urinary AcSDKP as an early biomarker in human CKD. We also thoroughly discussed the pharmacokinetic challenges of AcSDKP (e.g., rapid degradation by ACE, extremely short half-life) and proposed feasible clinical dosing regimens, such as leveraging ACE inhibitors to elevate endogenous AcSDKP or developing nanoparticle delivery systems. Modifications can be found in the Discussion section (Page 38, Line 594).

3.The discussion section is lengthy. A tighter focus on the novel mechanistic insights would be useful to readers and enhance impact.

Response: We completely agree. We have entirely rewritten and condensed the Discussion section. Redundant background information has been removed. The revised Discussion is now tightly focused on the dual role of AcSDKP, the FGFR1 paradox (incorporating the new siRNA data), model limitations, and clinical pharmacokinetics, making it much more concise and impactful.

Part II: Response to Reviewer #2

Reviewer #2 Summary: The text is mostly confusing... I request that the authors rewrite the text following the guidelines... A crucial segment is missing: Statistical analysis!... I still couldn’t understand the animal experiment... Finally, the authors used ether as method of euthanasia...

Response: We sincerely appreciate the reviewer’s strict and highly detailed scrutiny of our manuscript. Your rigorous standards have helped us identify and correct several critical flaws in our presentation, methodology description, and ethical documentation. We have implemented a massive overhaul of the manuscript to address every single concern you raised.

1. Text is confusing and excessively long. Rewrite the text following guidelines, eliminate redundancy. Submit the text to a specialist in the English language. Response: We deeply apologize for the confusing structure and redundant text in the original draft. We have systematically restructured and significantly shortened the Introduction and Discussion sections, eliminating irrelevant background information. The entire manuscript has undergone professional English language polishing to ensure clarity, proper flow, and adherence to journal guidelines.

2.A crucial segment is missing: Statistical analysis! Which statistical analysis did the authors use? Did the authors use any normality test?

Response: We sincerely apologize for omitting this crucial section. We have now added a comprehensive "Statistical Analysis" subsection in the Materials and Methods. Data are presented as mean ± standard deviation (SD) from at least three independent experiments. Normality of the data distribution was assessed using the Shapiro-Wilk test. For comparisons between two independent groups, an unpaired two-tailed Student’s t-test was performed. For comparisons among three or more groups, a One-way Analysis of Variance (ANOVA) followed by Tukey’s post hoc test was employed. In cases where the data did not pass the normality test, the non-parametric Mann-Whitney U test (for two groups) or Kruskal-Wallis test followed by Dunn's multiple comparisons test (for multiple groups) was utilized. All statistical analyses and graph generation were performed using GraphPad Prism 9.0 software (GraphPad Software, San Diego, CA, USA). A P-value < 0.05 was considered statistically significant. Modifications can be found in the Materials and Methods section (Page 14, Line 257).

3.Bioinformatics data: I couldn’t find anything about that in the Material and methods. Is this database available?

Response: We appreciate this important reminder. We have added a dedicated "Bioinformatics and RNA-Sequencing" section to the Materials and Methods. Furthermore, we have uploaded our raw sequence data to the Genome Sequence Archive (GSA) at the National Genomics Data Center. The accession number (CRA038202) and a statement of data availability have been prominently included. Modifications can be found in the Materials and Methods section (Page 13, Line 245).

4.Regarding antibodies used: missing companies, catalog numbers, and concentrations.

Response: We have meticulously updated the "Western Blot Analysis" and "Histology and Immunofluorescence" sections. All primary and secondary antibodies are now listed with their respective providing companies, specific catalog numbers, and working dilution concentrations. Modifications can be found in the Materials and Methods section (Page 11, Line 211).

5.RT-qPCR primers not mentioned. Disclose the primer sequence. Gene names should be in italics.

Response: Thank you for the correction. We have now provided all primer sequences in a newly added Table 1 and specified that they were commercially synthesized. Furthermore, we have carefully reviewed the entire manuscript and formatted all gene/mRNA names in italics (e.g., FGFR1, Hif1a) following standard nomenclature guidelines. Modifications can be found in Table 1 and throughout the manuscript.(Page 12, Line 232)

6.Animal experiment confusing (3 weeks, 4 weeks, before and after...). Was there any surgery involved? Anesthesia? Biopsy?

Response: We apologize for the previous confusing description. We have completely rewritten the "Animal Models and Experimental Design" section. To ensure absolute clarity, we separated the animal studies into two explicitly titled sub-models: 1) Time-Course Progression Model and 2) Self-Controlled Unilateral Intervention Model. We have clearly detailed that a complete "left nephrectomy was surgically performed" under anesthesia at specific timelines, rather than a biopsy, serving as the pre-treatment baseline. Modifications can be found in the Materials and Methods section (Page 6, Line 111). Flowchart of the experimental procedure for constructing a mouse aristolochic acid nephropathy model can be found in Figure 3a. (Figure 3a, Page 22, Line 351)

7.Euthanasia method: Ether is inhumane and alters physiological parameters. Disclose the animal protocol.

Response: We are extremely grateful for your vigilance regarding animal welfare. We deeply apologize for the documentation error in the original manuscript. The term "ether" was an inadvertent translation/typing error. The actual ethically approved protocol (Approval No. SUDA20240326A04) strictly utilized sodium pentobarbital. In the revised manuscript, we have corrected this and precisely detailed our ethically compliant procedures: Survival blood collection was performed via the tail vein, and terminal blood collection was performed via retro-orbital bleeding under deep sodium pentobarbital anesthesia, followed by cervical dislocation to confirm death, strictly adhering to AVMA and ARRIVE guidelines. Modifications can be found in the Materials and Methods section (Page 6, Line 111).

8.Figures: resolution, alignment, scale bars, sample size (n). The mention of a "trend" in Hif1a with n=3 is not fair.

Response: We sincerely appreciate your rigorous statistical critique. We fully agree that claiming a "trend" with a sample size of n=3 is scientifically inappropriate. To definitively address this concern and ensure the absolute reliability of our data, we have completely repeated the RT-qPCR experiment for Hif1a with an expanded sample size of n=6. The updated, appropriately powered results have now been incorporated into the revised figure. The results are now described strictly based on the robust statistical findings of the n=6 cohort.Additionally, we have meticulously revised all figures to ensure optimal resolution, proper alignment, and the inclusion of clear scale bars. The precise sample size is now explicitly stated in every figure legend.Modifications can be found in the Results section (Page 19, Line 317) and the corresponding Figure legend.(Page 46, Line 764)

9. References: Lack proper reference (sub-Saharan Africa, scleroderma, lines 78, 82, 88, 95-99). Doubled refs, unavailable refs, unrelated refs (line 116, 132, 136). Response: We deeply appreciate your rigorous fact-checking and sincerely apologize for the inaccuracies and poor curation in the original reference list. Through a comprehensive rewrite of the Introduction and Discussion sections, we have permanently removed the unsupported statements regarding sub-Saharan Africa, scleroderma models, and all loosely related or inaccurate references (including those originally in lines 116, 132, and 136).

Furthermore, we have significantly elevated the quality and relevance of the literature cited in our revised Discussion. To robustly contextualize our new mechanistic and translational insights, we have integrated several highly relevant, peer-reviewed studies. For instance, we incorporated recent literature detailing the pha

Attachment Submitted filename: Response to Reviewers.docx

# Peer review: Decision Letter 1

**DOI:** 10.1371/journal.pone.0355409.r003

**Authors:** Doa'a Al-u'datt

22 May 2026

Dear Dr. Li,

Thank you for submitting your manuscript to PLOS ONE. After careful consideration, we feel that it has merit but does not fully meet PLOS ONE’s publication criteria as it currently stands. Therefore, we invite you to submit a revised version of the manuscript that addresses the points raised during the review process.

Please submit your revised manuscript by Jul 06 2026 11:59PM. If you will need more time than this to complete your revisions, please reply to this message or contact the journal office at plosone@plos.org. When you're ready to submit your revision, log on to https://www.editorialmanager.com/pone/ and select the 'Submissions Needing Revision' folder to locate your manuscript file.

- A letter that responds to each point raised by the academic editor and reviewer(s). You should upload this letter as a separate file labeled 'Response to Reviewers'.
- A marked-up copy of your manuscript that highlights changes made to the original version. You should upload this as a separate file labeled 'Revised Manuscript with Track Changes'.
- An unmarked version of your revised paper without tracked changes. You should upload this as a separate file labeled 'Manuscript'.

If you would like to make changes to your financial disclosure, please include your updated statement in your cover letter. Guidelines for resubmitting your figure files are available below the reviewer comments at the end of this letter.

If applicable, we recommend that you deposit your laboratory protocols in protocols.io to enhance the reproducibility of your results. Protocols.io assigns your protocol its own identifier (DOI) so that it can be cited independently in the future. For instructions see: https://journals.plos.org/plosone/s/submission-guidelines#loc-laboratory-protocols. Additionally, PLOS ONE offers an option for publishing peer-reviewed Lab Protocol articles, which describe protocols hosted on protocols.io. Read more information on sharing protocols at https://plos.org/protocols?utm_medium=editorial-email&utm_source=authorletters&utm_campaign=protocols.

As the corresponding author, your ORCID iD is verified in the submission system and will appear in the published article. PLOS supports the use of ORCID, and we encourage all coauthors to register for an ORCID iD and use it as well. Please encourage your coauthors to verify their ORCID iD within the submission system before final acceptance, as unverified ORCID iDs will not appear in the published article. *Only*  the individual author can complete the verification step; PLOS staff *cannot*  verify ORCID iDs on behalf of authors.

We look forward to receiving your revised manuscript.

Kind regards,

Doa'a G. F. Al-u'datt

Academic Editor

PLOS One

Journal Requirements:

If the reviewer comments include a recommendation to cite specific previously published works, please review and evaluate these publications to determine whether they are relevant and should be cited. There is no requirement to cite these works unless the editor has indicated otherwise.

Please review your reference list to ensure that it is complete and correct. If you have cited papers that have been retracted, please include the rationale for doing so in the manuscript text, or remove these references and replace them with relevant current references. Any changes to the reference list should be mentioned in the rebuttal letter that accompanies your revised manuscript. If you need to cite a retracted article, indicate the article’s retracted status in the References list and also include a citation and full reference for the retraction notice.

[Note: HTML markup is below. Please do not edit.]

Reviewers' comments:

Reviewer's Responses to Questions

**Comments to the Author**

Reviewer #1: All comments have been addressed

Reviewer #2: All comments have been addressed

**********

2. Is the manuscript technically sound, and do the data support the conclusions?

Reviewer #1: Yes

Reviewer #2: Yes

**********

3. Has the statistical analysis been performed appropriately and rigorously?

Reviewer #1: Yes

Reviewer #2: Yes

**********

4. Have the authors made all data underlying the findings in their manuscript fully available?

The PLOS Data policy

Reviewer #1: Yes

Reviewer #2: Yes

**********

5. Is the manuscript presented in an intelligible fashion and written in standard English?

Reviewer #1: Yes

Reviewer #2: Yes

**********

Reviewer #1: The authors have made a genuine effort to address my concerns, which I appreciate. I have no further comments.

Reviewer #2: I appreciate the authors’ response to the concerns raised by both reviewers. The text is much more comprehensible now.

I noticed that the text contains some symbols, like the long dash, that are very characteristic of LLM software. Was any kind of generative AI used for the production of the manuscript? If so, it is important that this is disclosed, including the extension of the use of it (was it for writing the text? Translating it? Reviewing grammar?)

I understood the authors chose the aristolochic acid model for it to be replicable to the in vitro models. I wonder how AcSDKP would behave in another model of kidney fibrosis, such as unilateral ureter obstruction or renal ischemia-reperfusion model.

**********

what does this mean?). If published, this will include your full peer review and any attached files.

If you choose “no”, your identity will remain anonymous but your review may still be made public.

**Do you want your identity to be public for this peer review?**  For information about this choice, including consent withdrawal, please see our Privacy Policy

Reviewer #1: No

Reviewer #2: No

**********

[NOTE: If reviewer comments were submitted as an attachment file, they will be attached to this email and accessible via the submission site. Please log into your account, locate the manuscript record, and check for the action link "View Attachments". If this link does not appear, there are no attachment files.]

To ensure your figures meet our technical requirements, please review our figure guidelines: https://journals.plos.org/plosone/s/figures

You may also use PLOS’s free figure tool, NAAS, to help you prepare publication quality figures: https://journals.plos.org/plosone/s/figures#loc-tools-for-figure-preparation.

NAAS will assess whether your figures meet our technical requirements by comparing each figure against our figure specifications.

# Author comment: Author response to Decision Letter 2

**DOI:** 10.1371/journal.pone.0355409.r004

28 May 2026

Dear Academic Editor and Reviewers,

We sincerely thank the Academic Editor and both reviewers for their constructive evaluation of our revised manuscript. We are grateful that both reviewers considered the previous concerns to have been addressed. In this minor revision, we have focused on the remaining issues raised by Reviewer #2. Specifically, we have added a dedicated disclosure statement on the use of artificial intelligence tools, in accordance with PLOS policy; carefully rechecked the manuscript language and removed unnecessary long-dash punctuation and other stylistic symbols that could distract readers and expanded the Discussion to acknowledge the need to validate AcSDKP in additional kidney fibrosis models, particularly unilateral ureteral obstruction (UUO) and renal ischemia-reperfusion injury (I/R).

All revisions have been incorporated into the marked manuscript and the clean revised manuscript.

Reviewer #1

Comment: All comments have been addressed.

Response: We sincerely thank the reviewer for the positive evaluation and for recognizing our efforts to address the previous concerns.

Reviewer #2

Comment 1: I appreciate the authors’ response to the concerns raised by both reviewers. The text is much more comprehensible now. I noticed that the text contains some symbols, like the long dash, that are very characteristic of LLM software. Was any kind of generative AI used for the production of the manuscript? If so, it is important that this is disclosed, including the extension of the use of it (was it for writing the text? Translating it? Reviewing grammar?)

Response: We thank the reviewer for raising this important point. We agree that transparency regarding the use of artificial intelligence tools is essential. During preparation of the revised manuscript, Grammarly (Grammarly, Inc.) was used only to assist with English-language polishing, grammar checking, and improvement of sentence clarity. No artificial intelligence tool was used to generate experimental data, analyze data, perform statistical analysis, create figures or images, select or fabricate references, or formulate the scientific hypotheses, interpretations, conclusions, or limitations of the study. All AI-assisted edits were manually checked and approved by the authors against the original experimental data and source materials. The authors take full responsibility for the accuracy, integrity, and originality of all manuscript content.

To address the reviewer’s concern about style, we carefully revised the manuscript language and replaced unnecessary long dashes and AI-like punctuation with standard scientific English punctuation, while retaining only conventional symbols required for scientific notation, ranges, and figure references. We have also added the following dedicated disclosure statement to the Materials and Methods section of the manuscript:

During the preparation of the manuscript, Grammarly (Grammarly, Inc.) was used solely to assist with English-language polishing, grammar checking, and improvement of sentence clarity. The tool was not used to generate experimental data, analyze data, perform statistical analyses, create figures or images, select references, or develop the scientific hypotheses, interpretations, conclusions, or limitations of the study. All AI-assisted language edits were manually reviewed and verified by the authors against the original experimental records and source materials. The authors take full responsibility for the accuracy, integrity, and originality of the final manuscript content.

Comment 2: I understood the authors chose the aristolochic acid model for it to be replicable to the in vitro models. I wonder how AcSDKP would behave in another model of kidney fibrosis, such as unilateral ureter obstruction or renal ischemia-reperfusion model.

Response: We appreciate this insightful suggestion. We selected the aristolochic acid (AA)-induced CKD model because it allows direct alignment between the in vivo injury context and the AA-exposed HK-2 tubular epithelial cell model used in our mechanistic experiments. This design helped us interpret the compartment-specific effects of AcSDKP on pericyte-endothelial crosstalk and tubular epithelial injury within a consistent toxin-induced CKD framework.

We agree, however, that CKD-related fibrosis is etiologically heterogeneous and that additional models are needed to determine whether our findings are generalizable. Previous studies using UUO models have reported anti-fibrotic effects of Ac-SDKP, including reduced extracellular matrix deposition, myofibroblast accumulation, macrophage infiltration, and profibrotic mediator expression. Therefore, we would expect AcSDKP to retain at least part of its anti-fibrotic and vascular-protective activity in obstructive fibrosis. Nevertheless, the tubular epithelial response in UUO or I/R may differ from that observed in AA nephropathy because these models involve distinct drivers, including obstruction-associated pressure injury, inflammatory recruitment, hypoxia-reoxygenation injury, mitochondrial dysfunction, and AKI-to-CKD transition mechanisms. Thus, the extent to which VEGF/FGFR1-mediated pericyte-endothelial repair and FGFR1-independent tubular apoptosis are conserved across models remains an important question for future investigation.

Accordingly, we have added a limitation and future-direction statement to the Discussion as follows:

Although the AA model was selected to align the in vivo injury context with our AA-exposed HK-2 model and to examine a toxin-induced CKD setting, we acknowledge that CKD-associated fibrosis is heterogeneous. Prior studies in UUO models have reported anti-fibrotic effects of Ac-SDKP, including reduced extracellular matrix deposition, myofibroblast accumulation, macrophage infiltration, and profibrotic mediator expression. Therefore, AcSDKP may also exert anti-fibrotic activity in obstructive nephropathy. However, the tubular stress response observed in our AA model may not fully extrapolate to UUO or renal ischemia-reperfusion injury, where obstruction-driven pressure injury, inflammatory recruitment, hypoxia-reoxygenation, and mitochondrial injury predominate. Future studies using UUO and I/R models are needed to determine whether the VEGF/FGFR1-mediated pericyte-endothelial repair and the FGFR1-independent tubular apoptotic response observed here are conserved across etiologically distinct forms of kidney fibrosis. (Line 573-586)

We thank the Academic Editor and the reviewers again for their constructive comments, which have helped us improve the transparency, clarity, and translational framing of the manuscript.

Best regards,

The Authors

Pediatric Clinical Research Institute of Soochow University, Pediatric Clinical Medical College of Soochow University

Department of Nephrology and Immunology, Children’s Hospital of Soochow University, Soochow

Attachment Submitted filename: Responses.docx

# Peer review: Decision Letter 2

**DOI:** 10.1371/journal.pone.0355409.r005

**Authors:** Hiroyasu Nakano

21 Jul 2026

A Dual Role of AcSDKP in Chronic Kidney Disease: Targeting Pericyte-Driven Fibrosis and Vascular Repair via VEGF/FGFR1 While Eliciting a Compensatory FGFR1 Upregulation in Apoptotic Tubular Cells

PONE-D-25-54890R2

Dear Dr. Li,

We’re pleased to inform you that your manuscript has been judged scientifically suitable for publication and will be formally accepted for publication once it meets all outstanding technical requirements.

Within one week, you’ll receive an e-mail detailing the required amendments. When these have been addressed, you’ll receive a formal acceptance letter and your manuscript will be scheduled for publication.

An invoice will be generated when your article is formally accepted. Please note, if your institution has a publishing partnership with PLOS and your article meets the relevant criteria, all or part of your publication costs will be covered. Please make sure your user information is up-to-date by logging into Editorial Manager at Editorial Manager® and clicking the ‘Update My Information' link at the top of the page. For questions related to billing, please contact billing support.

If your institution or institutions have a press office, please notify them about your upcoming paper to help maximize its impact. If they’ll be preparing press materials, please inform our press team as soon as possible -- no later than 48 hours after receiving the formal acceptance. Your manuscript will remain under strict press embargo until 2 pm Eastern Time on the date of publication. For more information, please contact onepress@plos.org.

Kind regards,

Hiroyasu Nakano, M.D., Ph.D.

Academic Editor

PLOS One

Additional Editor Comments (optional):

Reviewers' comments:

Reviewer's Responses to Questions

**Comments to the Author**

Reviewer #1: All comments have been addressed

Reviewer #2: All comments have been addressed

**********

2. Is the manuscript technically sound, and do the data support the conclusions?

Reviewer #1: Yes

Reviewer #2: (No Response)

**********

3. Has the statistical analysis been performed appropriately and rigorously?

Reviewer #1: Yes

Reviewer #2: Yes

**********

4. Have the authors made all data underlying the findings in their manuscript fully available?

The PLOS Data policy

Reviewer #1: Yes

Reviewer #2: Yes

**********

5. Is the manuscript presented in an intelligible fashion and written in standard English?

Reviewer #1: Yes

Reviewer #2: Yes

**********

Reviewer #1: The authors have addressed my comments satisfactorily, I recommend acceptance of this manuscript at this stage.

Reviewer #2: I am satisfied with the response and I endorse the publicaiton of this manuscript! I appreciate the authors for the commitiment on running everything clear!

**********

what does this mean?). If published, this will include your full peer review and any attached files.

If you choose “no”, your identity will remain anonymous but your review may still be made public.

**Do you want your identity to be public for this peer review?**  For information about this choice, including consent withdrawal, please see our Privacy Policy

Reviewer #1: No

Reviewer #2: **Yes:** Bruno Paes Leme Ferreira

**********

# Editor report: Acceptance letter

**DOI:** 10.1371/journal.pone.0355409.r006

**Authors:** Hiroyasu Nakano

PONE-D-25-54890R2

PLOS One

Dear Dr. Li,

I'm pleased to inform you that your manuscript has been deemed suitable for publication in PLOS One. Congratulations! Your manuscript is now being handed over to our production team.

At this stage, our production department will prepare your paper for publication. This includes ensuring the following:

* All references, tables, and figures are properly cited

* All relevant supporting information is included in the manuscript submission,

* There are no issues that prevent the paper from being properly typeset

You will receive further instructions from the production team, including instructions on how to review your proof when it is ready. Please keep in mind that we are working through a large volume of accepted articles, so please give us a few days to review your paper and let you know the next and final steps.

Lastly, if your institution or institutions have a press office, please let them know about your upcoming paper now to help maximize its impact. If they'll be preparing press materials, please inform our press team within the next 48 hours. Your manuscript will remain under strict press embargo until 2 pm Eastern Time on the date of publication. For more information, please contact onepress@plos.org.

You will receive an invoice from PLOS for your publication fee after your manuscript has reached the completed accept phase. If you receive an email requesting payment before acceptance or for any other service, this may be a phishing scheme. Learn how to identify phishing emails and protect your accounts at https://explore.plos.org/phishing.

If we can help with anything else, please email us at customercare@plos.org.

Thank you for submitting your work to PLOS One and supporting open access.

Kind regards,

PLOS ONE Editorial Office Staff

on behalf of

Professor Hiroyasu Nakano

Academic Editor

PLOS One
